# Genetic affinities of the Jewish populations of India

**DOI:** 10.1038/srep19166

**Published:** 2016-01-13

**Authors:** Gyaneshwer Chaubey, Manvendra Singh, Niraj Rai, Mini Kariappa, Kamayani Singh, Ashish Singh, Deepankar Pratap Singh, Rakesh Tamang, Deepa Selvi Rani, Alla G. Reddy, Vijay Kumar Singh, Lalji Singh, Kumarasamy Thangaraj

**Affiliations:** 1 CSIR-Centre for Cellular and Molecular Biology, Hyderabad 500 007, India; 2 Department of Evolutionary Biology, Estonian Biocentre, Riia23b, Tartu, Estonia-51010; 3 Department of Anatomy, Amala Institute of Medical Sciences, Thrissur-680555, India; 4 Genome Foundation, Hyderabad 500007, India

## Abstract

Due to the lack of written records or inscription, the origin and affiliation of Indian Jewish populations with other world populations remain contentious. Previous genetic studies have found evidence for a minor shared ancestry of Indian Jewish with Middle Eastern (Jewish) populations. However, these studies (relied on limited individuals), haven’t explored the detailed temporal and spatial admixture process of Indian Jewish populations with the local Indian populations. Here, using large sample size with combination of high resolution biparental (autosomal) and uniparental markers (Y chromosome and mitochondrial DNA), we reconstructed genetic history of Indian Jewish by investigating the patterns of genetic diversity. Consistent with the previous observations, we detected minor Middle Eastern specific ancestry component among Indian Jewish communities, but virtually negligible in their local neighbouring Indian populations. The temporal test of admixture suggested that the first admixture of migrant Jewish populations from Middle East to South India (Cochin) occurred during fifth century. Overall, we concluded that the Jewish migration and admixture in India left a record in their genomes, which can link them to the ‘Jewish Diaspora’.

The Jewish communities are distributed in most parts of the world^1^, however, of all the Jewish Diaspora community, Indian Jewish are one of the least known people^2^,^3^,^4^. There are three main distinct groups of Jewish living in India (Fig. 1)- the Jewish of Cochin in Kerala, South India; the Bene Israel in Mumbai, West India and Baghdadi Jewish in Kolkata, East India^4^. Apart from these three distinct groups, there is fourth group known as Paradesi Jewish, who are supposed to be migrated from Portugal and Spain during 15-16^th^ Century and now integrated in to Cochin Jewish community^3^. It was observed that each of these communities are socially linked to their neighbours than one another^3^. There are several legendry stories about their migrations to India (Supplementary text), but because lack of written records and inscriptions, the origin and migrations of Indian Jewish remain moot^2^,^3^,^4^,^5^.

The Cochin Jewish are considered as the oldest and the first Jewish group appeared in India, who migrated during fifth or sixth century. Whereas the Bene Israel, the largest among all, were supposed to have migrated roughly one thousand years ago to the Maharashtra coast^2^,^3^,^5^,^6^. The recent newcomers were Baghdadi Jewish who migrated to East India during English regime (19^th^ Century)^7^. Yet, Indian Jewish are one of the early offshoot of Jewish Diaspora, their complex history, the presence of multiple subgroups on various regions of India, and persistence of ancestral social system, decorate them with several unique characteristics^2^,^3^,^4^,^6^,^7^.

Distinct to other Jewish communities^8^,^9^,^10^,^11^,^12^,^13^,^14^,^15^,^16^,^17^, Indian Jewish has not been the subject of extensive genetic study^11^,^18^,^19^,^20^. Nevertheless, being a distant outlier, the Indian Jewish have added a unique dimension to the Jewish Diaspora^11^,^18^,^19^. Previous genetic studies have utilised low resolution and limited samples from Cochin Jewish and Bene Israel, primarily as a reference population^11^,^16^,^20^. The first study on Cochin Jewish by utilising classical markers suggested their allele sharing both with Yemenite Jewish and South Indian populations^18^. Analysis of haploid and genomewide data on Cochin and Bene Israel communities, reported their clustering with the neighbouring autochthonous populations^11^,^19^. More specifically the mtDNA studies so far have identified largely South Asian autochthonous lineages among Jewish communities of India^11^,^19^, whereas the Y chromosomal investigation have reported both Indian and Middle Eastern specific lineages^11^. Recently, the Y chromosome analysis of Bene Israel linked them to Levant^20^, whilst the R1a-M582 clade frequent in Askenazi Jewish were absent among Indian Jewish^6^. All the above findings strongly indicate either high level of admixture of migrant Jewish with the local populations, or (and) religious integration of the local indigenous populations. Moreover, the extent of gene flow from Middle East, associated with the spread of Judaism in the India is still largely unknown. Therefore, to study the genetic signature and gain a better temporal and spatial understanding of their admixture with the native Indians, we present a detailed genetic characterization of the major Indian Jewish communities (Cochin Jewish and Bene Israel), by the combination of high resolution haploid and diploid genetic markers analysis.

## Results

### Autosomal SNP analysis

**Figure 1:**
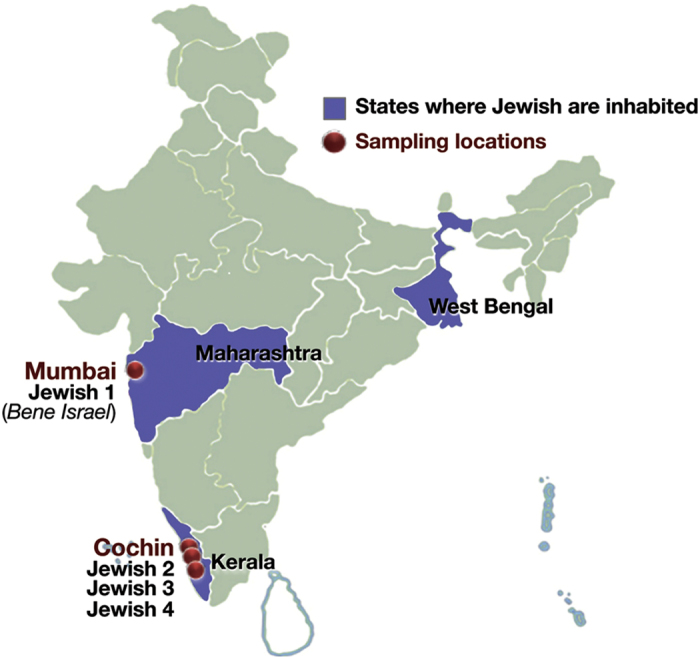
The settlement of Jewish populations in Indian Subcontinent. Indian Jewish samples collected in present study are highlighted by maroon dots. The map was modified from the Wikipedia (https://en.wikipedia.org/wiki/File:India-locator-map-blank.svg).

For the ease of understanding, we first classified different Indian Jewish groups present in our combined dataset (Supplementary Table 1). For autosomal analysis, we merged our data with the data coming from nine different studies^11^,^12^,^21^,^22^,^23^,^24^,^25^,^26^,^27^. The combined data was representing the Indian Jewish populations from two distinct geographical regions of India (Fig. 1). We renamed Bene Israel (Mumbai Jewish)^11^, coming from the West of India, to Jewish 1 and three South Indian Jewish groups^11^,^12^,^23^ as Jewish 2, Jewish 3 and Jewish 4 (Supplementary Table 1 and Supplementary text).

**Figure 2:**
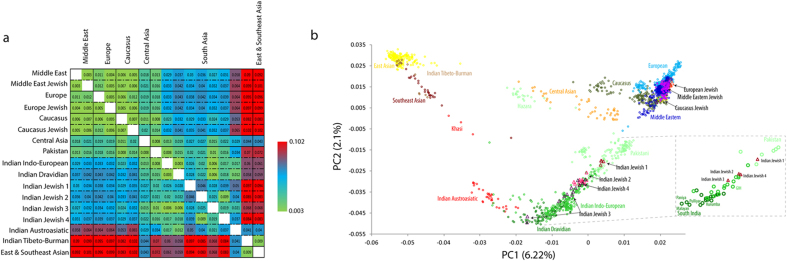
(**a)** Mean pairwise *F*st comparison of Indian Jewish with other regional populations obtained from the autosomal SNP data. (**b)** Principal component analysis *(PCA)* of the combined autosomal SNP data of individuals from Eurasia.

In the present study we have analysed samples from Cochin Jewish and Mumbai Jewish (Bene Israel) groups and referred them collectively as Indian Jewish (Fig. 1). To measure the genetic differentiation of Indian Jewish in terms of inter and intra-regional as well as at population level, we first calculated *F*st (Fig. 2a and Supplementary Table 2). The populationwise comparison analysis showed that the Indian Jewish share close affinity with their local South Asian neighbours, except for Indian Jewish 2 and Indian Jewish 4 (sampled from the same Indian Jewish territory) (Fig. 2a and Supplementary Table 2).

**Figure 3:**
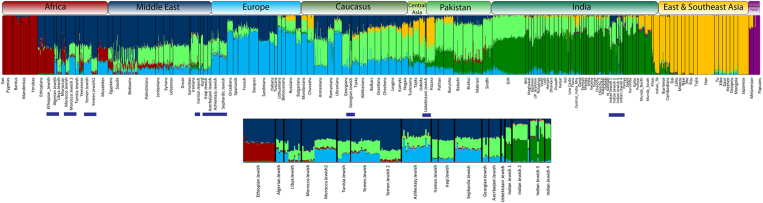
Results of the populationwise unsupervised ‘Structure-like’ ADMIXTURE analysis (*K* = 7) of world population projecting various Jewish populations.

We further used principle component analysis (PCA) to capture the genetic variation of Indian Jewish along the two axes covering the Eurasian landscape (Fig. 2b). Consistent with the *F*st analysis, the Indian Jewish were dispersed over the South Asian Indo-European-Dravidian cline. In agreement with the previous study^11^, the model based clustering method ADMIXTURE identified, three ancestral components among Indian Jewish populations (Fig. 3 and Supplementary Fig. 1). Supporting the *F*st and PCA results, the Indian ancestry was overwhelmingly dominant among Indian Jewish, however substantial traces of Middle Eastern ancestry (dark blue component) was also evident. The relative proportion of Middle Eastern component among Indian Jewish was observed between the range of 3–20% (Table 1). Conversely, the Middle Eastern ancestry was largely negligible among their neighbouring Indo-European and Dravidian populations. The spatial worldwide distribution of Middle Eastern (dark blue) component also showed elevation among Indian Jewish populations, which cannot be explained by the isolation by distance scenario, where one can expect the Middle Eastern ancestry gradient in India along the West-East and North-South axes (Supplementary Fig. 2). The Indian Jewish populations differ with each other in the context of harbouring the Near Eastern ancestry, as well as their placement in the PCA plot and *F*st variation (Fig. 2 and Supplementary Table 2). Therefore, regardless of the potential existence of a Near Eastern genetic link of Indian Jewish, substantial proportions of their genomes ancestry is shared with Indian populations.

To validate the Middle Eastern admixture in India Jewish populations, we have applied three (*f3*) and four (*f4*) population tests^23^,^25^,^28^. The outgroup *f3* statistics test showed significantly higher shared genetic drift of three Indian Jewish groups (Jewish 1, Jewish 2, Jewish 4), with the Middle Eastern, than their neighbouring local populations (Supplementary Fig. 3 and 4 and Table 2). Jewish 3 showed lowest affinity to Middle East in comparison with the rest Indian Jewish groups, nevertheless it was significantly higher than most of their neighbouring Dravidian populations (Table 2). The ANI (Ancestral North Indian) admixture proportion calculated from the *f4* ancestry test was consistently higher among Indian Jewish populations than their Indian neighbours (Supplementary Table 3). We also found higher number and longer length of segments of ROH among Indian Jewish 1 and Indian Jewish 4 groups, whilst the Indian Jewish 2 had lowest ROH segments among all the Indian Jewish populations (Supplementary Fig. 5).

**Figure 4:**
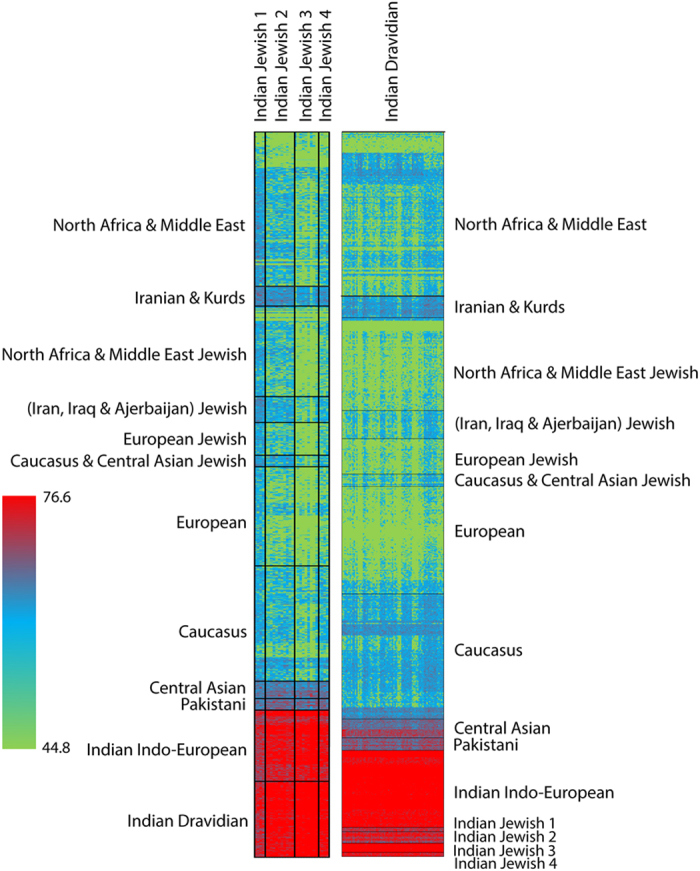
Comparison of the mean sharing of chunklengths of Indian Jewish vs Indian Dravidian neighbours received from Eurasian populations.

To identify the population structure of Indian Jewish based on haplotypes and recombination across the genome, we have further used ChromoPainter and performed fineSTRUCTURE analysis^29^. Consistent with the above analyses, all the Indian Jewish groups receive more number/length of chunks with local South Asian populations than their parental Middle Eastern populations (Fig. 4 and Table 3). Nevertheless, the neighbouring Dravidian local populations have significantly received lower number/length of Middle Eastern chunks in comparison with the Indian Jewish populations (two tailed p value <0.0001) (Table 3), which supports their (Indian Jewish) affinity with the Middle Eastern populations. Among all the four Jewish groups the attraction with the Middle Eastern ancestry was in Jewish 1 > Jewish 2 > Jewish 4 > Jewish 3 order (Fig. 4 and Table 3). Notably, we didn’t find any significant difference of chunk number/length sharing of Indian Jewish with Middle Eastern Jewish *vs*. non-Jewish populations (Table 3).

We applied LD based Alder method^30^, to estimate the time of admixture between Indian Jewish and their neighbouring local Indian populations. We have used Yemeni Jewish and Druze populations as Middle Eastern, while GIH, Paniya and Kurumba as local Indian surrogate populations (Supplementary Table 4). The Alder analysis of Indian Jewish 1 (by considering a generation time of 30 years), has yielded ~1100 years as the time of admixture with GIH population (Table 4). For three groups of Kerala Jewish (Jewish 2, Jewish 3 and Jewish 4), the time of admixture was oldest for Indian Jewish 4 (1590 years) whereas, Indian Jewish 3 showed a time of admixture of 1100 years. Surprisingly, the admixture time for Indian Jewish 2 was relatively recent (480 years).

### mtDNA and Y chromosomal analysis

**Figure 5:**
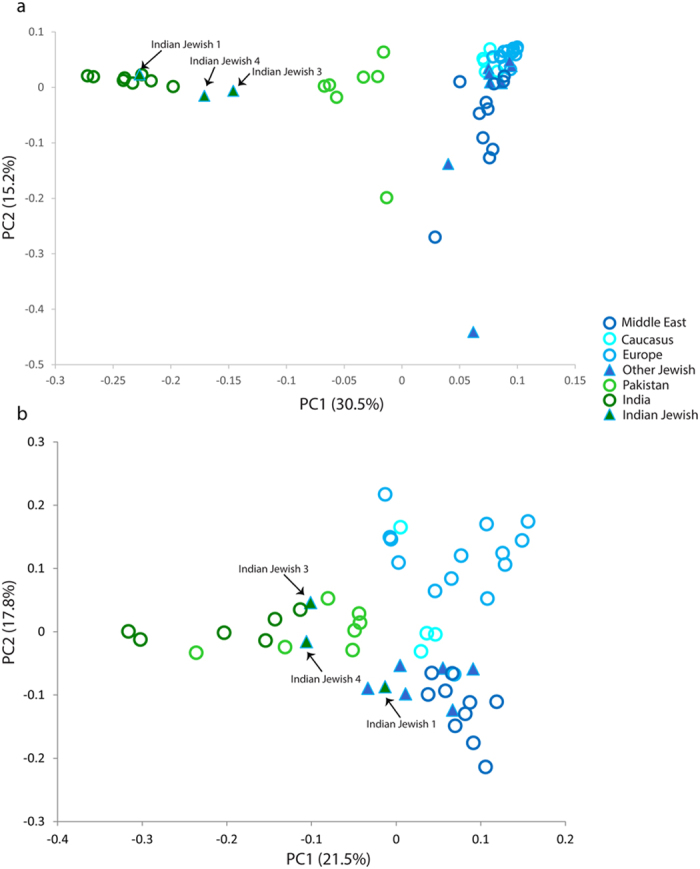
The placement of various Indian Jewish groups in the PC1 vs. PC2 analysis obtained from haplogroup frequencies for mtDNA; (a) and Y chromosome, (b) within other Eurasian populations.

To gain more insight about the sex specific Middle Eastern ancestry among Indian Jewish, we examined maternally inherited mitochondrial DNA (mtDNA) and paternally inherited Y chromosome biallelic markers in large sample sizes (Tables 5, 6 and Supplementary Tables 5,6). Consistent with the autosomal analysis, the mtDNA and Y chromosomal haplogroups were frequently South Asia specific (Tables 5 and 6). Apart from South Asian specific lineages (M2-6, M18, M30, M33, M35-37, M39-40, M64, N5, R5-6, R8, R30 and U2), the Indian Jewish also share 4.6% East Eurasian and 21.1% West Eurasian maternal lineages (Table 5). Among the West Eurasian lineages, subclades of haplogroup H, HV1, J, K, N1a and U5 were absent in their local neighbouring populations, which were otherwise predominant among Middle Eastern Jewish populations. (Table 5 and Supplementary Table 5). Interestingly, subclade K1a1b1a is also detected in Indian Jewish 3, which is one of the major founder lineage of the Jewish diaspora^8^,^17^, but was not observed among local Indian populations. The PC (Principle Component) analysis for mtDNA placed Indian Jewish 1 within the Indian cluster, whilst Jewish 3 and Jewish 4 were distracted away from the Indian core cluster because of higher proportion of genetic lineages of Middle East origin (Fig. 5a).

Similar to maternal haplogroup distribution, the paternal ancestry of Indian Jewish were also composed with some exclusive Middle East specific haplogroups (E,G, J(xJ2) and I) (Table 6). However, at the present level of resolution, it is not possible to link other common lineages (e.g. haplogroups J2 and R1a), which might have Middle Eastern roots (Table 6). The PC analysis was not well differentiated as in case of mtDNA, because of overwhelming presence of South Asian autochthonous lineages (Fig. 5b). Indian Jewish 3 and Jewish 4 clustered loosely to the South Asian knot, whereas Jewish 1 was located between Middle Eastern Jewish and South Asian populations (Fig. 5b).

## Discussion

According to the oral traditions, the first Jewish migrant to India arrived in South India (Kerala state), followed by Bene Israel in West India (Maharashtra), whereas Paradesi Jewish and Baghdadi Jewish in more recent times^2^,^3^,^4^,^6^,^7^. Genetic studies on classical markers as well as on uniparental and biparental markers have hitherto been found at some extant the Middle Eastern Genetic affinity of Indian Jewish populations^8^,^11^,^18^,^19^. However, the detailed analysis on the information about their origin, admixture and migration is largely lacking. Therefore, in this study by adding large number of Indian Jewish samples and groups, we reconstructed their history and showed that they have inherited their ancestry from Middle Eastern and Indian populations.

The population differentiation (*F*st) analysis suggested admixture of Indian Jewish with local Indian populations with some degree of relative isolation (Fig. 2a). The affinity with the local South Asian populations advocated their excessive admixture with the local Indian populations. All the Indian Jewish are far apart from each other except for the Jewish 2 and Jewish 4, who are closest to each other, likely because of sharing same geographical territory (Fig. 1).

Our result on ADMIXTURE analysis agrees on the presence of South Asian and Middle Eastern ancestral components among Indian Jewish populations (Fig. 3). Together with ADMIXTURE results, the PC analysis suggested overwhelming South Asia ancestry among Indian Jewish, responsible for their placement over the South Asian cline (Fig. 2a). Although we could not segregate Middle Eastern ancestry from the ANI (Ancestral North India), nonetheless it is one of the major denominator to elevate ANI ancestry among Indian Jewish populations, with respect to the local Indian populations (Supplementary Table 3). The Middle Eastern ancestry component over the geographical landscape of India is only well visible among Indian Jewish populations, whereas among local Indian populations it is largely absent (Tables 1, 2 and Supplementary Fig. 2). This argues against any major geneflow from the migrant Jewish populations towards their local neighbouring populations. However, the marked difference of effective population sizes of migrant Jewish and local Indian populations could easily dilute this signal in few generations.

The haplotype oriented analysis (Fig. 4) was in congruent with the allele frequency based analysis by showing minor (but significant) Middle Eastern signals in to the Indian Jewish populations. The time of admixture analysis has revealed that the first migration of Jewish in India (Cochin Jewish) has happened more than 1500 years ago, followed by the Bene Israel (Mumbai Jewish) roughly 1000 years ago (Table 4 and Supplementary Table 4). Therefore, the molecular time of admixture is largely consistent with the historical interpretations of Jewish migration in India^3^. Surprisingly, one of the South Indian Jewish group (Jewish 2) showed a recent time of admixture (480 years), which we can’t rule out as a migration of Paradesi Jewish group from Spain and Portugal. However, this group was more distant and didn’t share any excess of haplotypes with Sephardic Jewish than Middle Eastern populations (Figs. 2a, 4 and Supplementary Table 2).

For the sex-specific markers (mtDNA and Y chromosome), the distribution of various haplogroups are variable in each of the Indian Jewish group, not only when comparing with other Indian and world populations but also within the Indian Jewish (Tables 5, 6 and Supplementary Tables 5, 6). In a recent study it was suggested that one of the major maternal founder of European Jewish (haplogroup K1a1b1a) lineage has likely assimilated in Europe^17^. Surprisingly, its minor presence in Cochin Jewish (Jewish 3) group, who don’t show any recent European influx is intriguing (Table 5 and Supplementary Tables 5). The introgression of haplogroup K1a1b1a motif to Jewish 3 may have been either transmitted through Paradesi Jewish or via Middle East during the initial settlement.

The combined results of uniparental and biparental markers didn’t find any Middle Eastern specific signal in to the local Indian populations, suggesting that the direction of geneflow or population assimilation was largely unidirectional i.e. from local Indians to Indian Jewish. These results also invoke that the admixture of migrant Jewish with local Indian populations was not a continuous process. It was mainly driven by the religious conversion of the local populations. In case of prolonged geneflow, the ROH segments of Indian Jewish should have been very similar to the neighbouring Indian populations, which is not the case here (Supplementary Fig. 5). Moreover, the reduced diversity among Indian Jewish in all the genetics system testifies this scenario.

Overall, our pooled analysis of genetic variation among various groups of Indian Jewish populations, involving high-resolution sex-linked and autosomal markers, provides traces of Middle Eastern ancestry together with more likely unidirectional geneflow/admixture from their contemporary Indian populations. However, the Indian Jewish carry overwhelmingly South Asian ancestry, and the proportion of Middle Eastern genetic ancestry was minor, regardless of the genetic system explored. Moreover, sharing of specific mtDNA and Y chromosomal haplogroups between all the studied Indian Jewish and their abscence among other local Indian populations can be seen as a remnant of shared ancestry with Middle Eastern populations. The molecular data supports the model of migration of the Indian Jewish from Middle East, followed by the religious conversion and admixture with the local South Asian populations. The extensive admixture and assimilation can be seen clearly in our autosomal analysis with a rapid loss of Middle Eastern signals over the timeline. Nevertheless the rooted ancestry to their ancestral place can be testified because of a higher proportion of genetic lineages of Middle East origin.

## Material and Methods

### Sampling

About 5–10 ml blood samples with informed written consent were collected from 305 Cochin Jewish and 302 individuals from their seven neighbouring local populations (Kurchian, Ulladan, Malayan, Adiyan, Paniya, Kuruman and Kattunaikan), belonging to southern state Kerala of India (Fig. 1). With detailed interview procedure we have avoided individuals related minimum to three generations. This project was carried out in accordance with the approved guidelines and also permitted by the Institutional Ethical Committee of the Centre for Cellular and Molecular Biology-CSIR, Hyderabad, India. All experimental protocols were also approved by the Committee of the Centre for Cellular and Molecular Biology-CSIR, Hyderabad, India.

### Sample grouping

Since four different Jewish groups (coming from two geographical locations) have been analysed in this study, we have numbered them and used those numbers throughout the manuscript. We named Jewish 1 to Bene Israel (Mumbai Jewish) coming from West part of India. Jewish 1 data is extracted from Behar *et al*.^11^. Rest of the three Jewish group from South India are named as Jewish 2, Jewish 3 and Jewish 4. Jewish 2 is from Atzmon *et al*.^12^. Jewish 3 is our collection from Cochin (Kerala) and Jewish 4 is Cochin Jewish published in Behar *et al*.^11^. The details of SNPs and populations have been mentioned in Supplementary Table 1.

### Genotyping

We sequenced the Hypervariable segment I (HVS-I) of mtDNA by Sanger Sequencing method, utilising 23F and 23R markers described by Reider *et al*.^31^ Variations were scored the against the r-CRS^32^ and Reconstructed Sapiens Reference Sequence (RSRS)^33^. We further genotyped coding region diagnostic mutations and assigned their haplogroup based on combined information (Supplementary Tables 5 and 6). They were further confirmed by genotyping the coding regions mutations published till date in PhyloTree build 16^34^. For Y chromosome analysis, we genotyped more than 40 biallelic markers published elsewhere^35^ to assign the haplogroups among male individuals (Table 6). For all the markers, we have used direct Sanger sequencing method and assembled it with the reference to mark the variation. For autosomal genotyping, we used 15 Indian Jewish samples on Affymetrix (SNP 6.0) arrays by using standard protocols. We removed duplicate samples and filtered out individuals having less than 99% genotyping^36^. To include all the Indian Jewish populations we have merged the data published in nine different studies^11^,^12^,^21^,^22^,^23^,^24^,^25^,^26^,^27^. The merged data has yielded 98189 SNPs after quality control which we have used in our statistical analysis (Supplementary Table 1).

### mtDNA and Y-chromosome analysis

For mtDNA and Y chromosome analysis, we ran the Principle Component Analysis (PCA), using POPSTR (kindly provided by H. Harpending), to infer the relationship of populations based on haplogroup frequencies.

### Autosomal analysis

We have used different numbers of populations and datasets for various analyses (Supplementary Table 1). A check for closely related individuals was carried out within each study population by calculating average IBS (identity by state) scores for all pairs of individuals^36^. First, we sought to investigate the extent of population structure and admixture for Indian Jewish, embedded in their autosomal genomes.

We used PLINK 1.07^36^, to filter the combined dataset to include only SNPs on the 22 autosomal chromosomes with minor allele frequency >1% and genotyping success >99%. Because background linkage disequilibrium (LD) can affect both PCA^37^ and “Structure-like” (ADMIXTURE) analysis^38^, we thinned the dataset by removing one SNP of any pair in strong LD r^2^ > 0.4 in a window of 200 SNPs (sliding the window by 25 SNPs at a time). The pruned dataset has yielded 75594 SNPs (Supplementary Table 1).

We first calculated mean pairwise *F*_st_ differences between different population groups using the method of Cockerham and Weir^39^. Next we carried out PC analysis on pruned data using smartpca program (with default settings) of the EIGENSOFT package^37^to capture genetic variability described by the first 5 PCs. The fraction of the total variation described by a PC is the ratio of its eigenvalue to the sum of all eigenvalues. In the final setting we ran ‘Structure-like’ unsupervised ADMIXTURE, with a random seed number generator, on the LD pruned dataset twenty-five times at K = 2 to K = 12. Because the top values of the resulting log-likelihood scores were stable (virtually identical) within the runs of each K from K = 2 to K = 7 we can with some confidence argue, that convergence at global maximum was reached. The loglikelihood values showed seven ancestral populations as the best K value. Thus we omitted runs at K = 10 to K = 12 from further analysis. We have plotted the worldwide geographic distribution of Middle Eastern specific ancestry by utilising geographic coordinates of the studied populations by using Surfer 8 of Golden Software (Golden Software Inc., Golden, Colorado), following the Kriging procedure.

From the result of PC analysis we have removed two outlier samples of Indian Jewish 3 in further population based analysis. The outgroup *f*3^28^ statistics (Table 2 and Supplementary Fig. 3) was calculated by taking Paniya population (which was attaining outlier position among Indian Dravidians), as an outgroup *f*3 = (Paniya; Druze/Yemen Jewish, X), where X was Indian Jewish and their neighbouring local population. Subsequently, we have plotted the *f*3 results for shared drift with Middle East Jew outlier (Yemen Jewish) *vs*. Indian outlier (South Munda) (Supplementary Fig. 4). The ANI (Ancestral North Indian) ancestry was calculated using *f4* ancestry estimation test^28^ test (Supplementary Table 3). The Runs of Homozygosity (ROH) was calculated using Plink (Supplementary Fig. 5). For ROH calculations, we applied 1000kb windows size, a minimum of 100 SNPs per window allowing one heterozygous and five missing calls per window. For haplotype based analysis (fineSTRUCTURE)^29^, samples were phased with Beagle 3.3.2^40^. A coancestry matrix was constructed using ChromoPainter^29^ with the default settings. The mean chunk lengths of Indian Jewish with other populations was estimated. To estimate the admixture time we used the ALDER software^30^, between Indian Jewish and local Indian populations. From Middle Eastern side we have used Yemen Jewish as well as Druze populations as parental for Indian Jewish, whereas from Indian side, we have used different regional populations. For Jewish 1 (Bene Israel) we had only GIH (Gujarati) as surrogate, whilst for Jewish 2, Jewish 3 and Jewish 4 (all from South India), we have used Kurumba and Paniya populations (Table 4 and Supplementary Table 4).

## Additional Information

**How to cite this article**: Chaubey, G. *et al*. Genetic affinities of the Jewish populations of India. *Sci. Rep*. **6**, 19166; doi: 10.1038/srep19166 (2016).

## Supplementary Material

Supplementary Information

## Figures and Tables

**Table 1:** The Middle Eastern specific ancestry (%) among Indian populations inferred from ADMIXTURE.

Group	Middle East Ancestry (+SD)
Indian Indo-European	0.68 ± 0.99
Indian Dravidian	0.90 ± 0.98
Indian Jewish 1	19.77 ± 1.14
Indian Jewish 2	11.70 ± 1.46
Indian Jewish 3	2.86 ± 1.42
Indian Jewish 4	10.34 ± 1.21

**Table 2:** Shared drift (*f3*) analysis results suggesting Middle Eastern affinity for Indian Jewish populations.

*f3* = (Paniya; Yemen_Jewish,X)			*f3* = (Paniya; Druze,X)		
X = North India Populations	*f3*	SD	X = North India Populations	*f3*	SD
GIH	4.21 × 10^−2^	0.11 × 10^−2^	GIH	4.35 × 10^−2^	0.11 × 10^−2^
Bhil	3.07 × 10^−2^	0.12 × 10^−2^	Bhil	3.18 × 10^−2^	0.12 × 10^−2^
Indian Jewish 1	5.47 × 10^−2^	0.15 × 10^−2^	Indian Jewish 1	5.51 × 10^−2^	0.15 × 10^−2^
**X = South India Populations**			**X = South India Populations**		
Kurumba	2.92 × 10^−2^	0.11 × 10^−2^	Kurumba	2.99 × 10^−2^	0.11 × 10^−2^
Sakilli	2.98 × 10^−2^	0.12 × 10^−2^	Sakilli	3.08 × 10^−2^	0.12 × 10^−2^
Indian Jewish 2	4.65 × 10^−2^	0.12 × 10^−2^	Indian Jewish 2	4.70 × 10^−2^	0.12 × 10^−2^
Indian Jewish 3	4.12 × 10^−2^	0.12 × 10^−2^	Indian Jewish 3	4.16 × 10^−2^	0.13 × 10^−2^
Indian Jewish 4	4.59 × 10^−2^	0.14 × 10^−2^	Indian Jewish 4	4.65 × 10^−2^	0.15 × 10^−2^

**Table 3:** The average Chunk Lengths and Chunk Counts donated by different population groups to various Indian Jewish groups.

Group (receiver)	Chunk Length donor				Chunk Count donor			
	Middle East Jewish	Middle East non Jewish	Indian Indo-European	Indian Dravidian	Middle East Jewish	Middle East non Jewish	Indian Indo-European	Indian Dravidian
Indian Jewish 1	66.9 ± 1.49	66.74 ± 2.26	72.24 ± 2.03	72.92 ± 1.96	53.6 ± 0.59	53.72 ± 1.17	56.93 ± 0.9	57.43 ± 0.78
Indian Jewish 2	65.58 ± 1.29	65.29 ± 1.95	74.29 ± 2.26	75.37 ± 2.03	53 ± 0.54	53.01 ± 1.15	57.96 ± 0.96	58.68 ± 0.8
Indian Jewish 3	63.6 ± 1.36	63.98 ± 1.92	76.01 ± 2.62	78.33 ± 2.98	51.88 ± 0.55	52.3 ± 1.14	58.94 ± 1.26	60.28 ± 1.28
Indian Jewish 4	65.41 ± 1.41	65.38 ± 2.15	74.18 ± 2.15	75.45 ± 1.93	52.77 ± 0.53	52.99 ± 1.18	57.94 ± 1.01	58.75 ± 0.81
Indian Dravidian neighbours	62.88 ± 1.35	63.28 ± 1.88	78.39 ± 3.11	82.57 ± 9.87	51.43 ± 0.54	51.83 ± 1.12	60.19 ± 1.5	62.45 ± 3.48

**Table 4:** The time of admixture of Jewish with local Indian populations, inferred from the Alder analysis.

Admixed population	Surrogate population 1	Surrogate population 2	Generations of Admixture (SD)	p value	Z score
Indian Jewish 1	GIH	Druze	37 (9)	6.9 × 10^−5^	3.98
Indian Jewish 2	Kurumba	Druze	16 (5)	5 × 10^−4^	3.48
Indian Jewish 3	Kurumba	—	37 (11)	1.6 × 10^−4^	3.23
Indian Jewish 4	Kurumba	Druze	53 (11)	3.1 × 10^−5^	4.17

**Table 5:** The maternal haplogroup sharing of West Indian (Maharashtra), South Indian (Kerala), Indian Jewish and other World Jewish populations.

Group	*n*	A	B	C	D	F	H	HV	HV1	HV2	I	J	K	L	M	M1	N	N1a	N1b	N1c	N5	R	R0a	Z	T	U	U1	U2	U3	U4	U5	U6	U7	U8	W	X	Reference
West Indian	269	—	—	0.022	—	—	—	0.019	—	—	—	—	—	—	0.610	—	—	—	—	—	0.011	0.167	—	—	—	0.004	0.011	0.119	—	—	—	—	0.026	—	0.011	—	16, 41
South Indian	485	—	—	0.014	—	—	—	—	—	—	—	—	—	—	0.827	—	—	—	—	—	0.002	0.087	—	—	—	—	0.010	0.031	—	0.023	—	—	0.006	—	—	—	11, Present Study
Indian Jewish 1	34	—	—	—	—	—	0.088	—	—	—	—	—	—	—	0.824	—	—	—	—	—	—	0.088	—	—	—	—	—	—	—	—	—	—	—	—	—	—	19
Indian Jewish 3	305	0.007	0.016	0.003	—	0.003	—	—	0.023	—	—	0.013	0.026	—	0.400	—	—	0.003	—	—	0.007	0.249	—	0.026	—	—	0.049	0.118	—	0.003	0.010	—	0.043	—	—	—	Present Study
Indian Jewish 4	52	—	—	0.135	0.019	—	—	—	—	—	—	—	—	—	0.442	—	—	0.038	—	—	—	0.231	—	—	—	—	0.115	—	—	—	—	—	—	—	0.019	—	19
Other World Jewish	1308	0.001	—	—	—	0.001	0.232	0.033	0.041	0.008	0.022	0.110	0.181	0.040	0.005	0.011	0.005	0.001	0.051	0.001	—	0.016	0.031	—	0.078	0.002	0.017	0.004	0.019	0.008	0.015	0.006	0.015	0.005	0.019	0.026	11, 16, 19

**Table 6:** The paternal haplogroup sharing of West Indian (Maharashtra), South Indian (Kerala), Indian Jewish and other World Jewish populations.

Group	*n*	E-M35	C3-M217	C-M130	F-M89	G-M201	H-M69	I-M170	J(xJ2)	J2-M172	K-M9	L-M11	N-M214	O-M175	PQR2	R-M207	R1-M173	R1a-M198	Reference
West Indian Brahmin	96	—	—	0.03	0.02	—	0.15	—	—	0.08	0.02	0.14	—	—	0.14	0.02	—	0.41	42, 43, 44
Marathi (West Indian)	36	—	—	0.06	0.03	—	0.33	—	—	0.19	—	0.11	—	—	0.14	—	—	0.14	43, 44
South Indian	284	—	0.01	0.12	0.25	—	0.19	—	—	0.05	—	0.05	—	0.01	0.15	0.01	0.01	0.15	11, Present Study
Indian Jewish 1	31	0.06	—	0.03	—	0.06	0.16	—	0.19	0.42	—	—	—	—	—	—	—	0.06	11
Indian Jewish 3	162	—	0.01	0.01	0.05	0.05	0.12	0.02	—	0.07	0.04	0.08	0.02	0.04	0.09	0.03	0.01	0.36	Present Study
Indian Jewish 4	45	—	—	0.04	0.02	—	—	0.02	—	0.20	—	0.16	—	—	0.42	0.04	—	0.09	11
Other World Jewish	1155	0.18	—	—	0.01	0.08	0.01	0.03	0.22	0.18	0.03	—	—	—	0.08	0.09	—	0.10	11
