# Corrigendum: Genetic affinities of the Jewish populations of India

**DOI:** 10.1038/srep26421

**Published:** 2016-05-23

**Authors:** Gyaneshwer Chaubey, Manvendra Singh, Niraj Rai, Mini Kariappa, Kamayani Singh, Ashish Singh, Deepankar Pratap Singh, Rakesh Tamang, Deepa Selvi Rani, Alla G. Reddy, Vijay Kumar Singh, Lalji Singh, Kumarasamy Thangaraj

Scientific Reports 6: Article number: 1916610.1038/srep19166; published online: 01132016; updated: 05232016.

After publication of our paper, it was kindly brought to our attention that the genomewide dataset (11 samples) for the Indian Jewish2 group in its entirety is unpublished. It was accidentally shared with us as a part of the data we obtained from authors of Atzmon *et al*. (ref. 12). Hence, in this Corrigendum, we exclude all analyses and interpretations made based on the Indian Jewish2 group from our paper. These data will be included as a part of a future publication (Yedael Y. Waldman, Arjun Biddanda, Maya Dubrovsky, Christopher L. Campbell, Carole Oddoux, Eitan Friedman, Gil Atzmon, Eran Halperin, Harry Ostrer, and Alon Keinan, *The genetic history of Cochin Jews from India*, manuscript in submission). The removal of this dataset and the inferred results neither alters our conclusions about other Indian Jewish groups (Jewish1, Jewish3 and Jewish4), nor affect our main findings. We apologize for this error and thank Drs. Gil Atzmon, Alon Keinan, and Harry Ostrer for bringing this to our attention.

