# Supplementary material for: Genetic affinities of the Jewish populations of India
Source: Sci Rep. 2016 Jan 13;6:19166. doi: 10.1038/srep19166 (PMC4725824; doi:10.1038/srep19166)
Supplement: Supplementary Information [file srep19166-s1.pdf]

Supplementary Text

Genetic affinities of the Jewish populations of India

Gyaneshwer Chaubey [1,2§], Manvendra Singh [1§], Niraj Rai [1§], Mini Kariappa [1,3§], Kamayani Singh [1], Ashish Singh [1], Deepankar Pratap Singh [1], Rakesh Tamang [1], Deepa Selvi Rani [1], Alla G Reddy [1], Vijay Kumar Singh [1], , Lalji Singh [1,4] and Kumarasamy Thangaraj [1] *

[1]*CSIR-Centre for Cellular and Molecular Biology, Hyderabad 500 007, India*

[2]*Department of Evolutionary Biology, Estonian Biocentre, Riia23b, Tartu, Estonia-51010*

[3] *Department of Anatomy, Amala Institute of Medical Sciences, Thrissur-680555, India*

[4] *Genome Foundation, Hyderabad 500007, India*

§ These authors contributed equally to this work

Extended data analysis

Autosomal analysis

First, we sought to investigate the extent of population structure and admixture among the Indian Jewish and other Eurasian populations embedded in their autosomal genomes. We used Affymatrix 6.0 genotyping chips on our collection of Indian Jewish (Cochin Jewish) sample and genotyped 10 individuals. These results were combined with the global data set of 1865 individuals generated with Affymatrix 6.0, Illumina 610K, 650K chips (For a full list of populations and sample sizes see Supplementary Table 1). After excluding SNPs unique to either of these platforms and SNPs from mtDNA, X and Y chromosomes the combined data set had data for 98189 SNPs that was used in subsequent analyses. We have excluded single outlier of Indian Jewish 1 and two outlier samples of Indian Jewish 3 for the population based *F*st, *f3, f4* and Runs of Homozygosity (ROH) analysis. We thinned the dataset by removing one SNP of a pair in strong LD $r^2 > 0.4$ in a window of 200 SNPs (sliding the window by 25 SNPs at a time). Finally we were left with a data matrix of 75594 SNPs.

mtDNA and Y chromosome

The collection of our samples were from Indian Jewish 3 (Cochin Jewish). The Indian Jewish 1 and Indian Jewish 4 data on haploid DNA analysis were published elsewhere[1-4]. For the Indian Jewish 2 the haploid DNA data was not available. For the mtDNA genotyping, we have sequenced first Hypervariable segment I (HVS-I) and diagnostic coding region mutations by utilizing the primers published in Reider et al. [5]. Individuals with ambiguous haplogroup affiliation were subjected to further sequencing of informative coding region stretches. Similarly, for the Y chromosome genotyping we have used defining SNPs and the published primers to segregate samples in to the major subclades [6,7]. Sequencing of mtDNA and Y chromosome markers was carried out in ABI 3730 and 3730XL DNA analyzers. To minimize errors both strands were double-sequenced. Sequences were assembled, and edited using Autoassembler2.0. Mutations were scored

relative to the r-CRS and RSRS [8,9]. All the mutations were confirmed by manual checking of their electropherograms.

Extended history of Indian Jewish

India is inhabited by several indigenous populations and there too have been several historical migrations [10,11]. Jewish are one of the recent migrants and they have been found in various part of India [12]. The migrations of Jewish populations to India is poorly understood. One of the views is that ten Jewish Families released from Persian Jail by the king in BC605 have arrived Kodungallore, Kerala, India now called as Cochin Jewish group. However, their arrival in India has not been well documented and any contemporary scholars argue their arrival at some time during the early Middle Ages. [12-14].

India harbors <5% of world Jewish population [15,16]. Among the major communities of Indian Jewish populations, the Cochin Jewish community was thought to be the pioneer among the Indian Jewish who entered to India [12,17]. The Bene Israel was considered as second group which has entered to the Konkan area (Maharashtra state) through sea from Middle East around 100 AD In India [12,13]. The Paradesi Jewish group arrived to Malabar coast (Kelara) during 15-16th Century after their exicle from Spain [13,14,17]. More recently the Baghdadi Jewish group from modern Iran, Iraq and adjoining region migrated to Kolkata (East of India) during end of 18th Century [13,18,19].

In the contemporary India the Paradesi Jewish community are integrated with Cochin Jewish and also many Jewish groups are converted to Christianity. Jewish were just another group, not subject to particular exclusion or marginalization. Non-Jewish could, and did, move into the Indian Jewish community, while this was taboo in the Islamic or Christian world [12,13,15,17,18]. Bene Israel is the largest group among Indian Jewish community, followed by Cochin Jewish and Baghdadi Jewish [16].

Because of different population histories and affiliation with various diverse Middle Eastern groups, the relation between different Indian Jewish are rather complex. It was shown that the Indian Jewish communities hardly ever formed a social contact and common identity [19].

References

1. Metspalu, M. *et al.* Most of the extant mtDNA boundaries in south and southwest Asia were likely shaped during the initial settlement of Eurasia by anatomically modern humans. *BMC Genet.* **5**, 26 (2004).

2. Behar, D. M. *et al.* The matrilineal ancestry of Ashkenazi Jewry: portrait of a recent founder event. *Am J Hum Genet.* **78**, 487-497 (2006).

3. Behar, D. M. *et al.* Counting the founders: the matrilineal genetic ancestry of the Jewish Diaspora. *PLoS ONE.* **3**, e2062 (2008).

4. Behar, D. M. *et al.* The genome-wide structure of the Jewish people. *Nature.* **466**, 238-242 (2010).

5. Rieder, M. J., Taylor, S. L., Tobe, V. O. & Nickerson, D. A. Automating the identification of DNA variations using quality-based fluorescence re-sequencing: analysis of the human mitochondrial genome. *Nucleic Acids Res.* **26**, 967-73. (1998).

6. YCC A nomenclature system for the tree of human Y-chromosomal binary haplogroups. *Genome Res.* **12**, 339-348 (2002).

7. Karafet, T. M. *et al.* New binary polymorphisms reshape and increase resolution of the human Y chromosomal haplogroup tree. *Genome Res.* **18**, 830-838 (2008).

8. Andrews, R. M. *et al.* Reanalysis and revision of the Cambridge reference sequence for human mitochondrial DNA. *Nat Genet.* **23**, 147 (1999).

9. Behar, D. M. *et al.* A "Copernican" reassessment of the human mitochondrial DNA tree from its root. *The American Journal of Human Genetics.* **90**, 675-684 (2012).

10. Chaubey, G. The demographic history of India: A perspective based on genetic evidence (http://hdl.handle.net/10062/15240). PhD. (Universitatis Tartuensis, Estonia, 2010).

11. Romila, T. A History of India, Vol. 1.

12. Slapak, O. *The Jews of India* (UPNE, 1995).

13. Katz, N. *Who Are the Jews of India?* (Univ of California Press, 2000).

14. Israel, R. R. *The Jews of India* , 2002).

15. Fernandes, E. *The Last Jews Of Kerala* (Granta Books, 2011).

16. Ehrlich, M. A. *Encyclopedia of the Jewish Diaspora* (ABC-CLIO, 2009).

17. Roland, J. G. *The Jewish Communities of India* (Transaction Publishers, 1998).

18. Ṿail, S. *India's Jewish heritage* (Marg Publications, 2002).

19. Egorova, Y. *Jews and India* (Psychology Press, 2006).

Present affiliation:

Manvendra Singh: Max Delbrueck Center for Molecular Medicine (MDC), Berlin, 13125, Germany. E mail- mnujnu@gmail.com

Kamayani Singh: University of Oxford, Wellcome Trust Centre for Human Genetics, University of Oxford, Oxford OX3 7BN UK. E mail- singh.kamayani@gmail.com

Deepankar Pratap Singh: Friedrich Miescher Institute for Biomedical Research Maulbeerstrasse 66 4058 Basel, Switzerland. E mail- deepankar.singh@fmi.ch

Ashish Singh: Toxicogenomics and Predictive Toxicology lab, CSIR-Indian Institute of Toxicology Research (IITR) Lucknow, India ashish222985@gmail.com

Rakesh Tamang: Department of Zoology, University of Calcutta, Kolkata 700019, India. E mail- rakesh.tamang7@gmail.com

Vijay Kumar Singh: Dept. of Pediatric Hematology/Oncology, Justus-Liebig. University Giessen, Feulgenstraße 12, 35392 Giessen, Germany. E mail- vijayvishen@gmail.com

Legend for Supplementary Figures:

Supplementary Fig. 1. The ADMIXTURE plot for K=2-9.

Supplementary Fig. 2. The spatial distribution of Middle Eastern specific ancestry component obtained from ADMIXTURE analysis. The map was generated by using Surfer8 of Golden Software (Golden Software Inc., Golden, Colorado).

Supplementary Fig. 3. The plot of shared drift obtained by the f3 =(Yoruba; Druze, X). The f3 values were plotted on Y axis against the X- targeted populations on X axis.

Supplementary Fig. 4. Comparison of Middle East vs Indian ancestry sharing of various Eurasian populations, obtained from f3 shared drift analysis.

Supplementary Fig. 5. The average count of number and length of ROH (Runs of Homozygosity) segments among various populations including Indian Jewish.

Supplementary Fig. 1

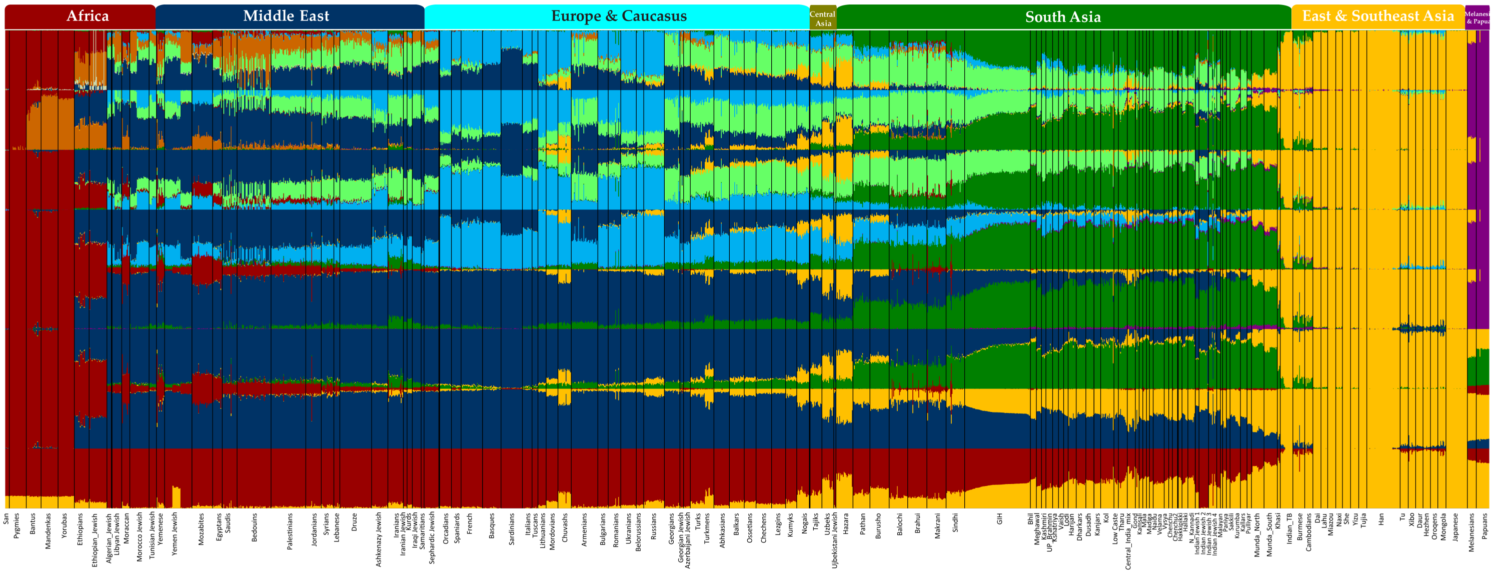

Supplementary Fig. 2

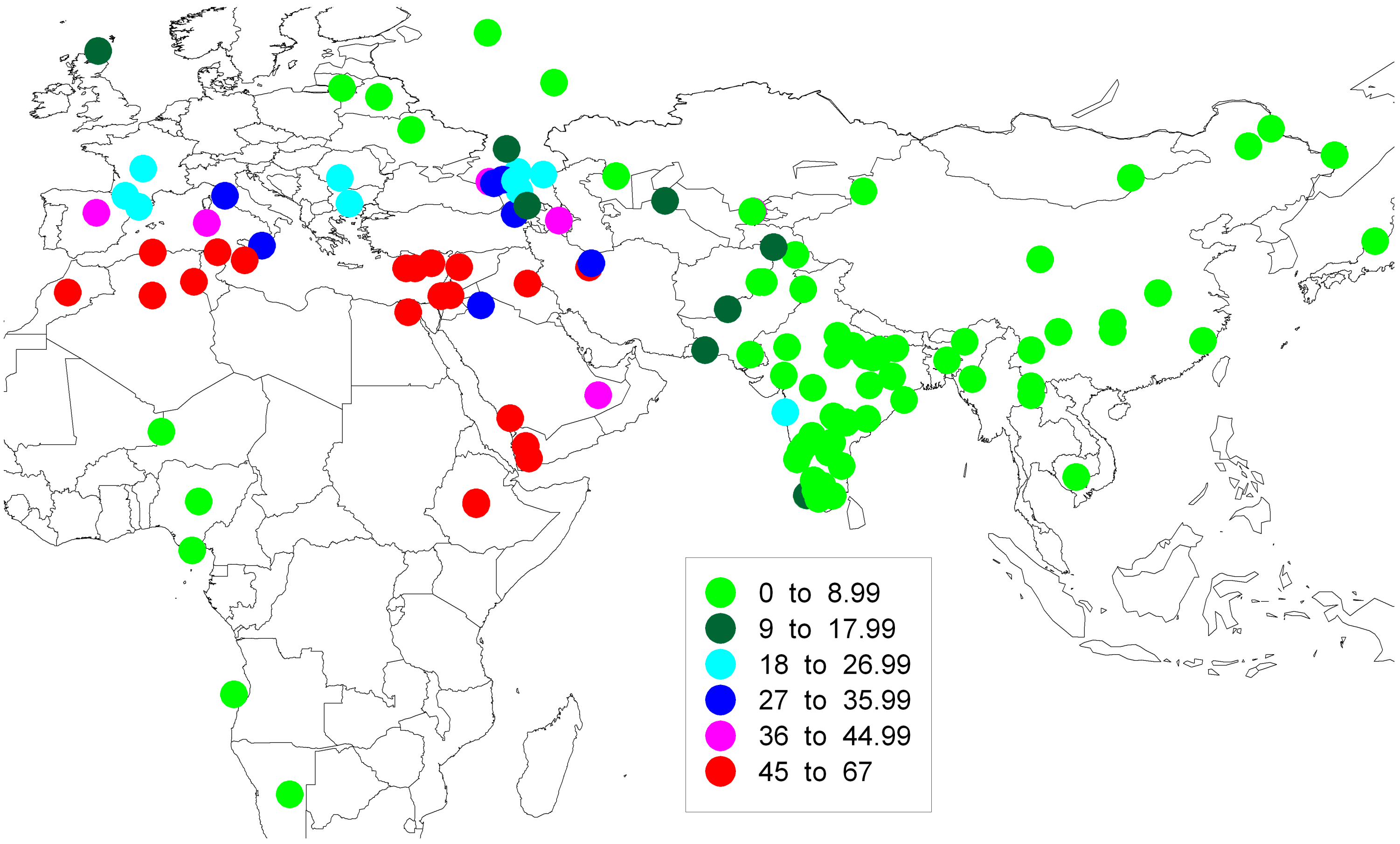

Supplementary Fig. 3

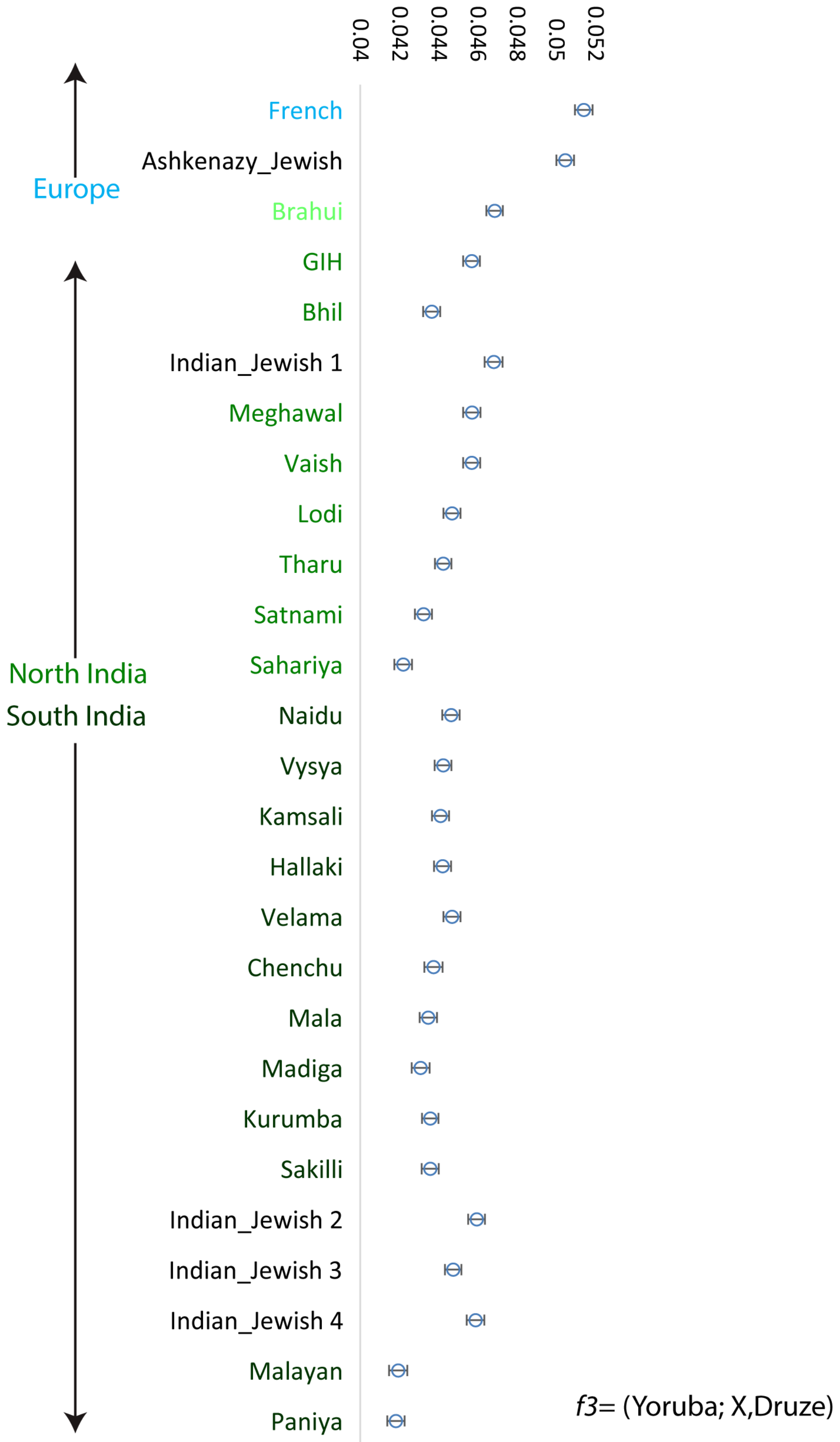

Supplementary Fig. 4

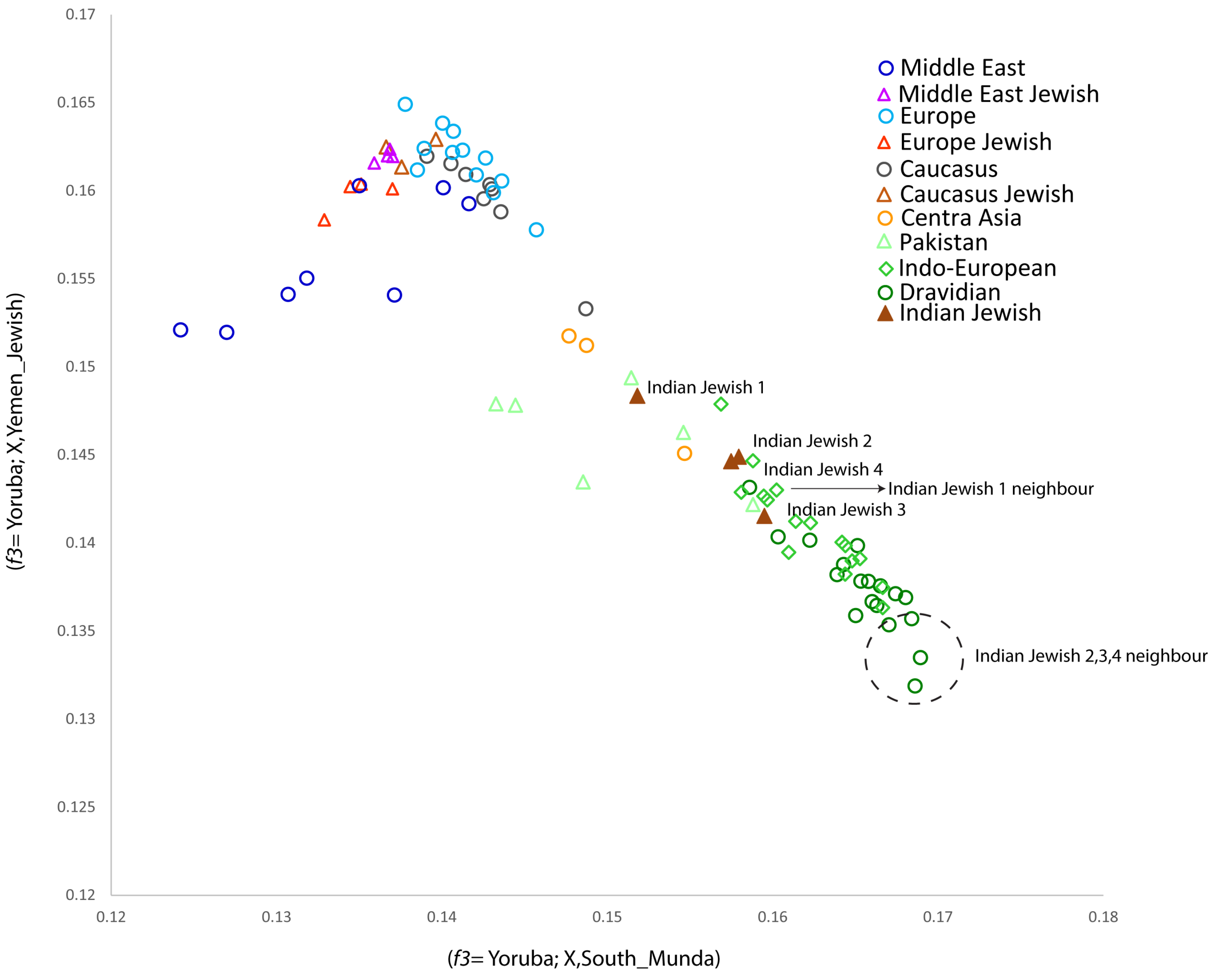

Supplementary Fig. 5

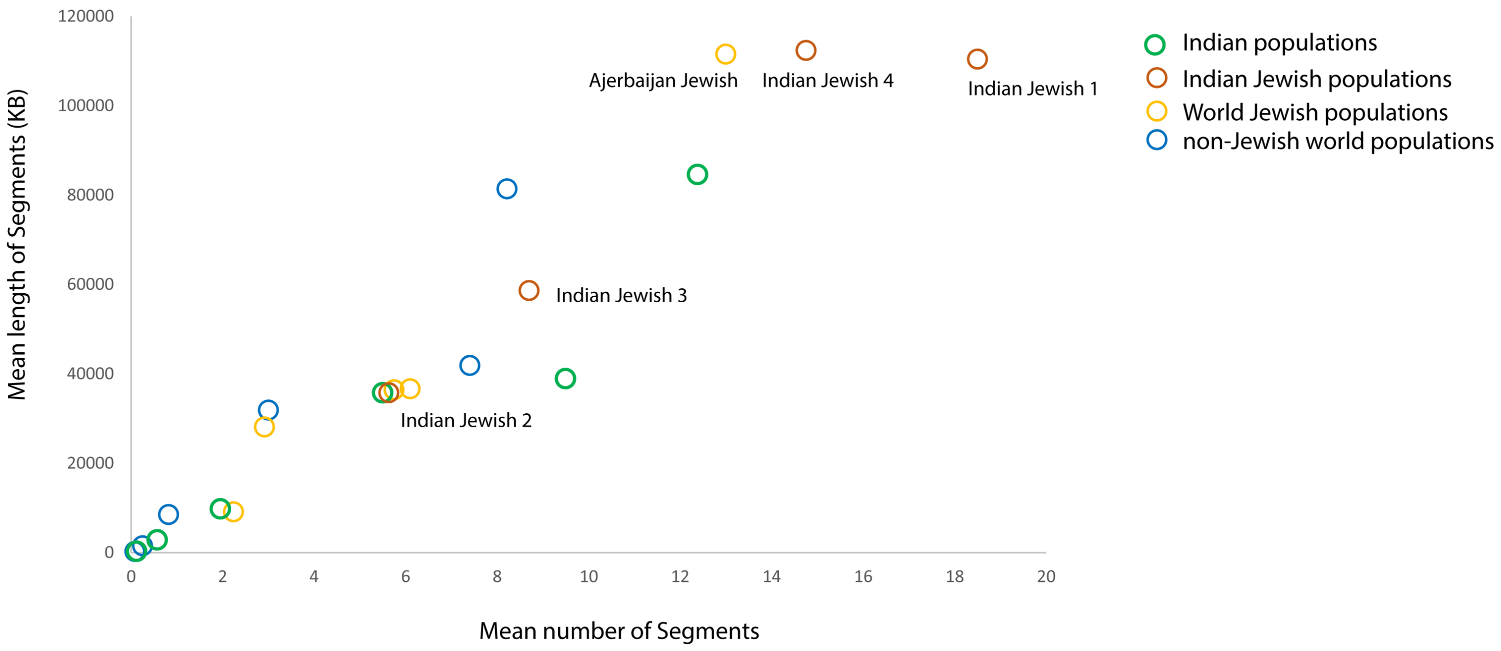

Supplementary Table 1. The details of the populations and number of samples, used in various autosomal analysis

Region	Population	n (ADMIXTURE)	n (PCA)	n (fineStructure)	n (f3, f4, ROH)	Reference
Africa	San	5	0	0	5	Li et al. 2008
Africa	Pygmies	22	0	0	22	Li et al. 2008
Africa	Bantus	19	0	0	19	Li et al. 2008
Africa	Mandenkas	22	0	16	22	Li et al. 2008
Africa	Yorubas	21	0	15	21	Li et al. 2008
Africa	Ethiopians	19	0	14	19	Behar et al. 2010
Africa	Ethiopian Jewish	23	0	18	23	Behar et al. 2010
Africa	Mozabites	27	0	14	27	Li et al. 2008
Africa	Algerian Jewish	5	0	5	5	Behar et al. 2010
Africa	Libya Jewish	12	0	12	12	Behar et al. 2010
Africa	Moroccans	10	0	7	10	Behar et al. 2010
Africa	Morocco Jewish	25	0	18	25	Behar et al. 2010
Africa	Tunisia Jewish	9	0	9	9	Behar et al. 2010
Middle East	Yemenese	10	10	10	10	Behar et al. 2010, Atzmon et al. 2010
Middle East	Yemen Jewish	36	12	20	36	Behar et al. 2010, Atzmon et al. 2010
Middle East	Egyptans	12	0	12	12	Behar et al. 2010, Atzmon et al. 2010
Middle East	Saudis	19	18	14	19	Behar et al. 2010, Atzmon et al. 2010
Middle East	Bedouins	45	45	0	45	Li et al. 2008
Middle East	Palestinians	46	46	17	46	Li et al. 2008
Middle East	Jordanians	20	19	15	20	Behar et al. 2010, Atzmon et al. 2010
Middle East	Syrians	16	16	16	16	Behar et al. 2010, Atzmon et al. 2010
Middle East	Lebanese	7	7	7	7	Behar et al. 2010, Atzmon et al. 2010
Middle East	Druze	42	42	16	42	Li et al. 2008
Middle East	Iranians	20	20	14	20	Behar et al. 2010
Middle East	Iranian Jewish	4	4	4	4	Behar et al. 2010, Atzmon et al. 2010
Middle East	Kurds	6	6	6	6	Behar et al. 2010
Middle East	Iraqi Jewish	11	20	15	11	Behar et al. 2010, Atzmon et al. 2010
Middle East	Samaritians	3	3	3	3	Behar et al. 2010
Europe	Ashkenazy Jewish	21	21	13	21	Behar et al. 2010, Atzmon et al. 2010
Europe	Sephardic Jewish	19	24	19	19	Behar et al. 2010, Atzmon et al. 2010
Europe	Orcadians	15	15	0	15	Behar et al. 2010
Europe	Spaniards	12	12	12	12	Behar et al. 2010
Europe	French	28	28	13	28	Li et al. 2008
Europe	Basques	24	24	0	24	Li et al. 2008
Europe	Sardinians	28	28	12	28	Li et al. 2008
Europe	Italians	12	12	12	12	Li et al. 2008
Europe	Tuscans	7	7	7	7	Li et al. 2008
Europe	Lithuanians	10	10	0	10	Behar et al. 2010
Europe	Belorussians	9	9	0	9	Behar et al. 2010
Europe	Russians	27	27	13	27	Li et al. 2008
Caucasus	Mordovians	15	15	15	15	Yunusbayev et al. 2011
Caucasus	Chuvashs	17	17	14	17	Yunusbayev et al. 2011
Caucasus	Armenians	35	35	0	35	Behar et al. 2010, Yunusbayev et al. 2011
Caucasus	Bulgarians	13	13	13	13	Yunusbayev et al. 2011
Caucasus	Romanians	16	16	13	16	Yunusbayev et al. 2011
Caucasus	Ukranians	20	20	10	20	Yunusbayev et al. 2011
Caucasus	Georgians	20	20	14	20	Behar et al. 2010, Yunusbayev et al. 2011
Caucasus	Georgian Jewish	4	10	10	4	Behar et al. 2010
Caucasus	Azerbaijani Jewish	8	8	8	8	Behar et al. 2010
Caucasus	Turks	19	19	13	19	Yunusbayev et al. 2011
Caucasus	Abhkasians	20	20	0	20	Yunusbayev et al. 2011
Caucasus	Balkars	19	19	14	19	Yunusbayev et al. 2011
Caucasus	N_Ossetians	15	15	0	15	Yunusbayev et al. 2011
Caucasus	Chechens	20	20	12	20	Yunusbayev et al. 2011
Caucasus	Lezgins	18	18	13	18	Behar et al. 2010, Yunusbayev et al. 2011
Caucasus	Kumyks	14	14	14	14	Yunusbayev et al. 2011
Caucasus	Nogais	16	16	11	16	Yunusbayev et al. 2011
Central Asia	Tajiks	15	15	10	15	Yunusbayev et al. 2011
Central Asia	Turkmens	12	12	0	12	Yunusbayev et al. 2011
Central Asia	Uzbeks	15	15	8	15	Behar et al. 2010
Central Asia	Uzbekistani Jewish	2	2	2	2	Behar et al. 2010
South Asia	Hazara	22	22	0	22	Li et al. 2008
South Asia	Pathan	22	22	0	22	Li et al. 2008
South Asia	Burusho	25	25	0	25	Li et al. 2008
South Asia	Balochi	24	24	12	24	Li et al. 2008
South Asia	Brahui	25	25	0	25	Li et al. 2008
South Asia	Makrani	25	24	0	25	Li et al. 2008
South Asia	Sindhi	24	23	0	24	Li et al. 2008
South Asia	GIH	88	88	11	88	HapMap3
South Asia	Bhil	7	7	7	7	Reich et al. 2009
South Asia	Rajasthan	6	6	6	6	Reich et al. 2009, Metspalu et al. 2011
South Asia	Kashmiri	5	5	0	5	Reich et al. 2009
South Asia	Brahmins_UP	8	8	8	8	Metspalu et al. 2011
South Asia	Kshatriya	7	7	7	7	Metspalu et al. 2011
South Asia	Vaish	6	4	4	6	Reich et al. 2009
South Asia	Lodi	5	5	0	5	Reich et al. 2009
South Asia	Chamar	10	10	10	10	Metspalu et al. 2011
South Asia	Dharkars	12	12	0	12	Metspalu et al. 2011
South Asia	Dusadh	10	10	10	10	Metspalu et al. 2011
South Asia	Kanjars	8	8	0	8	Metspalu et al. 2011
South Asia	Kol	17	17	14	17	Metspalu et al. 2011
South Asia	Low Caste_UP	5	5	0	5	Metspalu et al. 2011
South Asia	Tharu	11	11	0	11	Reich et al. 2009, Metspalu et al. 2011
South Asia	Central_India_mix	10	10	0	10	Metspalu et al. 2011
South Asia	Malayan	2	2	2	2	Behar et al. 2010
South Asia	Paniya	4	4	4	4	Behar et al. 2010
South Asia	Sakilli	4	4	4	4	Behar et al. 2010
South Asia	Kurumba	13	13	13	13	Reich et al. 2009, Metspalu et al. 2011
South Asia	P_Kallars	8	8	8	8	Metspalu et al. 2011
South Asia	Pulliyar	5	5	5	5	Metspalu et al. 2011
South Asia	Gond	4	4	4	4	Metspalu et al. 2011
South Asia	Kamsali	4	4	0	4	Reich et al. 2009
South Asia	Madiga	4	4	4	4	Reich et al. 2009
South Asia	Mala	3	3	3	3	Reich et al. 2009
South Asia	Naidu	4	4	4	4	Reich et al. 2009
South Asia	Velama	14	14	10	14	Reich et al. 2009, Metspalu et al. 2011
South Asia	Vysya	5	5	5	5	Reich et al. 2009
South Asia	Chenchu	4	4	0	4	Metspalu et al. 2011
South Asia	Chenchu2	6	6	0	6	Reich et al. 2009
South Asia	Hakkipikki	4	4	4	4	Metspalu et al. 2011
South Asia	Hallaki	7	7	0	7	Metspalu et al. 2011
South Asia	Kannadi	9	9	0	9	Behar et al. 2010
South Asia	Indian Jewish 1 (Bene Israel)	4	4	4	4	Behar et al. 2010
South Asia	Indian Jewish 2 (Cochin Jewish)	11	11	10	10	Atzmon et al. 2010
South Asia	Indian Jewish 3 (Cochin Jewish)	10	10	9	8	Moorjani et al. 2013; Present Study
South Asia	Indian Jewish 4 (Cochin Jewish)	4	4	4	4	Behar et al. 2010
South Asia	Munda North	16	16	0	16	Chaubey et al. 2011
South Asia	Munda South	17	17	0	17	Chaubey et al. 2011
South Asia	Khasi	3	3	0	3	Chaubey et al. 2011
South Asia	Indian_TB	16	16	0	16	Reich et al. 2009, Metspalu et al. 2011
Southeast Asia	Burmese	15	15	0	15	Chaubey et al. 2011
Southeast Asia	Cambodians	10	10	0	10	Li et al. 2008
East Asia	Dai	10	10	0	10	Li et al. 2008
East Asia	Lahu	8	8	0	8	Li et al. 2008
East Asia	Miaozu	10	10	0	10	Li et al. 2008
East Asia	Naxi	8	8	0	8	Li et al. 2008
East Asia	She	10	10	0	10	Li et al. 2008
East Asia	Yizu	10	10	0	10	Li et al. 2008
East Asia	Tujia	10	10	0	10	Li et al. 2008
East Asia	Han	44	44	0	44	Li et al. 2008
East Asia	Tu	10	10	0	10	Li et al. 2008
East Asia	Xibo	9	9	0	9	Li et al. 2008
East Asia	Daur	9	9	0	9	Li et al. 2008
East Asia	Hezhen	9	9	0	9	Li et al. 2008
East Asia	Oroqens	9	9	0	9	Li et al. 2008
East Asia	Mongola	10	10	0	10	Li et al. 2008
East Asia	Japanese	28	28	0	28	Li et al. 2008
PNG	Melanesians	11	0	0	11	Li et al. 2008
PNG	Papuans	17	0	0	17	Li et al. 2008
	**Total Samples**	**1875**	**1606**	**778**	**1872**	
	**Total SNPs after QC**	**75594**	**75594**	**98189**	**75594**	

Li, J. Z. *et al.* Worldwide human relationships inferred from genome-wide patterns of variation. *Science.* **319**, 1100-1104 (2008).

Behar, D. M. *et al.* The genome-wide structure of the Jewish people. *Nature.* **466,** 238-242 (2010).

Atzmon, G. *et al.* Abraham's children in the genome era: major Jewish diaspora populations comprise distinct genetic clusters with shared Middle Eastern Ancestry. *Am J Hum Genet.* **86**, 850-859 (2010).

Yunusbayev, B. *et al.* The Caucasus as an Asymmetric Semipermeable Barrier to Ancient Human Migrations. *Mol Biol Evol.* (2011).

International HapMap 3 Consortium *et al.* Integrating common and rare genetic variation in diverse human populations. *Nature.* **467**, 52-58 (2010).

Reich, D., Thangaraj, K., Patterson, N., Price, A. L. & Singh, L. Reconstructing Indian population history. *Nature.* **461**, 489-494 (2009).

Metspalu, M. *et al.* Shared and unique components of human population structure and genome-wide signals of positive selection in South Asia. *Am J Hum Genet.* **89**, 731-744 (2011).

Moorjani, P. *et al.* Genetic evidence for recent population mixture in India. *Am J Hum Genet.* **93**, 422-438 (2013).

Chaubey, G. *et al.* Population Genetic Structure in Indian Austroasiatic speakers: The Role of Landscape Barriers and Sex-specific Admixture. *Mol Biol Evol.* **28**, 1013-1024 (2011).

Supplementary Table. 2. The populationwise pairwise *F* st caculated from autosomal data

	Yemenese	Bedouins	Druze	Iranians	Jordanians	Kurds	Lebanese	Palestinians	Saudis	Syrians	Yemenite Jewish	Iranian Jewish	Iraqi Jewish	Syrian Jewish	Belorussians	Bulgarians	French
Yemenese	0	0.016	0.023	0.018	0.012	0.022	0.015	0.016	0.014	0.015	0.019	0.025	0.025	0.026	0.034	0.025	0.03
Bedouins	0.016	0	0.013	0.01	0.005	0.011	0.006	0.008	0.004	0.006	0.009	0.013	0.013	0.014	0.024	0.014	0.019
Druze	0.023	0.013	0	0.009	0.007	0.009	0.006	0.009	0.012	0.006	0.014	0.013	0.011	0.013	0.019	0.01	0.014
Iranians	0.018	0.01	0.009	0	0.004	0.002	0.003	0.007	0.009	0.002	0.012	0.008	0.008	0.011	0.014	0.007	0.011
Jordanians	0.012	0.005	0.007	0.004	0	0.005	9E-04	0.003	0.004	8E-04	0.007	0.007	0.007	0.009	0.016	0.007	0.012
Kurds	0.022	0.011	0.009	0.002	0.005	0	0.003	0.008	0.011	0.003	0.014	0.011	0.009	0.012	0.016	0.007	0.011
Lebanese	0.015	0.006	0.006	0.003	9E-04	0.003	0	0.004	0.005	0	0.009	0.007	0.008	0.01	0.015	0.006	0.011
Palestinians	0.016	0.008	0.009	0.007	0.003	0.008	0.004	0	0.007	0.003	0.009	0.011	0.01	0.011	0.018	0.01	0.013
Saudis	0.014	0.004	0.012	0.009	0.004	0.011	0.005	0.007	0	0.005	0.008	0.012	0.012	0.014	0.024	0.014	0.019
Syrians	0.015	0.006	0.006	0.002	8E-04	0.003	0	0.003	0.005	0	0.007	0.006	0.007	0.008	0.014	0.005	0.01
Yemenite Jewish	0.019	0.009	0.014	0.012	0.007	0.014	0.009	0.009	0.008	0.007	0	0.016	0.014	0.016	0.027	0.017	0.021
Iranian Jewish	0.025	0.013	0.013	0.008	0.007	0.011	0.007	0.011	0.012	0.006	0.016	0	0.01	0.017	0.025	0.013	0.018
Iraqi Jewish	0.025	0.013	0.011	0.008	0.007	0.009	0.008	0.01	0.012	0.007	0.014	0.01	0	0.011	0.022	0.012	0.016
Syrian Jewish	0.026	0.014	0.013	0.011	0.009	0.012	0.01	0.011	0.014	0.008	0.016	0.017	0.011	0	0.021	0.012	0.015
Belorussians	0.034	0.024	0.019	0.014	0.016	0.016	0.015	0.018	0.024	0.014	0.027	0.025	0.022	0.021	0	0.004	0.004
Bulgarians	0.025	0.014	0.01	0.007	0.007	0.007	0.006	0.01	0.014	0.005	0.017	0.013	0.012	0.012	0.004	0	0.001
French	0.03	0.019	0.014	0.011	0.012	0.011	0.011	0.013	0.019	0.01	0.021	0.018	0.016	0.015	0.004	0.001	0
French_Basques	0.037	0.025	0.021	0.019	0.019	0.02	0.018	0.02	0.026	0.017	0.028	0.026	0.023	0.021	0.012	0.009	0.007
Lithuanians	0.039	0.029	0.024	0.018	0.021	0.02	0.02	0.023	0.029	0.019	0.033	0.03	0.026	0.025	0.001	0.006	0.006
North_Italians	0.028	0.016	0.012	0.009	0.009	0.01	0.007	0.01	0.016	0.007	0.018	0.016	0.014	0.012	0.007	0.001	0.001
Orcadians	0.036	0.025	0.02	0.016	0.018	0.017	0.017	0.02	0.025	0.016	0.029	0.024	0.023	0.021	0.006	0.007	0.004
Romanians	0.025	0.015	0.011	0.007	0.007	0.007	0.006	0.01	0.015	0.006	0.017	0.014	0.012	0.012	0.004	0	0.002
Russians	0.035	0.026	0.021	0.015	0.018	0.016	0.017	0.02	0.026	0.016	0.029	0.024	0.023	0.022	0.002	0.005	0.005
Sardinians	0.035	0.021	0.017	0.018	0.015	0.02	0.014	0.016	0.021	0.014	0.023	0.024	0.02	0.018	0.017	0.009	0.009
Spaniards	0.028	0.016	0.013	0.01	0.01	0.011	0.009	0.011	0.016	0.008	0.019	0.018	0.015	0.013	0.006	0.002	5E-04
Tuscans	0.024	0.013	0.009	0.006	0.005	0.009	0.005	0.008	0.013	0.004	0.016	0.012	0.012	0.012	0.008	0.002	0.002
Ukranians	0.034	0.022	0.018	0.013	0.015	0.014	0.014	0.017	0.022	0.013	0.025	0.022	0.019	0.018	7E-04	0.002	0.003
Ashkenazy Jewish	0.024	0.013	0.01	0.008	0.008	0.009	0.007	0.009	0.013	0.006	0.015	0.014	0.012	0.011	0.012	0.005	0.007
Italian Jewish	0.021	0.009	0.01	0.008	0.003	0.008	3E-04	0.007	0.01	0.003	0.013	0.013	0.012	0.011	0.017	0.008	0.011
Sephardic Jewish	0.02	0.009	0.007	0.005	0.004	0.006	0.002	0.005	0.009	0.002	0.011	0.01	0.008	0.007	0.011	0.004	0.006
Abhkasians	0.026	0.016	0.011	0.006	0.009	0.007	0.008	0.011	0.015	0.006	0.018	0.014	0.012	0.013	0.015	0.008	0.011
Armenians	0.022	0.01	0.007	0.003	0.004	0.003	0.003	0.006	0.01	0.002	0.012	0.009	0.007	0.008	0.013	0.004	0.008
Balkars	0.026	0.016	0.012	0.005	0.009	0.006	0.007	0.011	0.016	0.006	0.019	0.013	0.012	0.014	0.011	0.006	0.009
Chechens	0.028	0.019	0.014	0.008	0.011	0.008	0.01	0.013	0.018	0.009	0.021	0.016	0.014	0.016	0.013	0.009	0.011
Chuvashs	0.036	0.03	0.026	0.017	0.022	0.019	0.021	0.024	0.029	0.02	0.034	0.028	0.028	0.027	0.011	0.012	0.013
Georgians	0.027	0.015	0.011	0.006	0.008	0.007	0.008	0.01	0.015	0.006	0.018	0.014	0.011	0.013	0.015	0.008	0.011
Kumyks	0.024	0.014	0.01	0.004	0.007	0.004	0.006	0.01	0.014	0.005	0.017	0.01	0.011	0.013	0.011	0.005	0.008
Lezgins	0.027	0.018	0.012	0.006	0.01	0.006	0.008	0.013	0.017	0.007	0.02	0.014	0.013	0.015	0.012	0.007	0.009
Mordovians	0.034	0.025	0.02	0.014	0.017	0.015	0.017	0.019	0.025	0.015	0.029	0.023	0.022	0.021	0.002	0.005	0.005
Nogais	0.026	0.019	0.016	0.007	0.011	0.009	0.01	0.014	0.018	0.009	0.022	0.016	0.016	0.017	0.012	0.008	0.011
North_Ossetians	0.027	0.017	0.013	0.006	0.009	0.008	0.009	0.012	0.016	0.007	0.02	0.015	0.013	0.015	0.013	0.007	0.011
Turks	0.021	0.011	0.007	0.002	0.004	0.002	0.002	0.006	0.01	0.002	0.013	0.007	0.008	0.009	0.01	0.004	0.007
Azerbaijani Jewish	0.03	0.019	0.016	0.013	0.012	0.014	0.011	0.015	0.018	0.011	0.02	0.017	0.017	0.019	0.025	0.016	0.02
Georgian Jewish	0.023	0.013	0.01	0.007	0.006	0.008	0.006	0.009	0.012	0.006	0.013	0.012	0.01	0.012	0.02	0.011	0.014
Hazara	0.043	0.041	0.038	0.026	0.032	0.029	0.032	0.036	0.039	0.03	0.044	0.038	0.039	0.041	0.035	0.032	0.035
Tajiks	0.026	0.02	0.016	0.006	0.012	0.008	0.01	0.015	0.019	0.01	0.023	0.015	0.017	0.019	0.014	0.011	0.013
Turkmens	0.028	0.021	0.018	0.009	0.013	0.01	0.013	0.016	0.02	0.011	0.024	0.018	0.019	0.021	0.02	0.014	0.017
Uzbeks	0.033	0.03	0.027	0.016	0.021	0.019	0.021	0.025	0.029	0.02	0.034	0.027	0.028	0.03	0.023	0.021	0.023
Balochi	0.025	0.021	0.018	0.007	0.013	0.008	0.011	0.016	0.019	0.011	0.024	0.015	0.017	0.02	0.02	0.015	0.02
Brahui	0.026	0.022	0.019	0.008	0.014	0.01	0.013	0.017	0.02	0.012	0.025	0.017	0.018	0.022	0.022	0.017	0.021
Burusho	0.035	0.031	0.027	0.015	0.022	0.018	0.022	0.025	0.03	0.02	0.035	0.027	0.027	0.03	0.026	0.023	0.025
Makrani	0.022	0.019	0.017	0.006	0.011	0.008	0.01	0.015	0.018	0.01	0.022	0.014	0.017	0.02	0.021	0.016	0.019
Pathan	0.028	0.023	0.019	0.007	0.014	0.01	0.013	0.018	0.022	0.012	0.026	0.016	0.019	0.022	0.018	0.015	0.017
Sindhi	0.028	0.025	0.023	0.01	0.017	0.012	0.016	0.02	0.024	0.015	0.028	0.019	0.022	0.024	0.022	0.019	0.022
Bhil	0.048	0.046	0.046	0.031	0.037	0.038	0.04	0.042	0.045	0.037	0.052	0.048	0.047	0.05	0.047	0.044	0.046
Brahmins_UP	0.035	0.03	0.028	0.015	0.022	0.019	0.021	0.025	0.029	0.019	0.035	0.028	0.028	0.031	0.026	0.024	0.025
Chamar	0.049	0.047	0.046	0.031	0.038	0.038	0.04	0.042	0.045	0.037	0.052	0.046	0.047	0.049	0.047	0.043	0.045
Dharkars	0.046	0.044	0.043	0.028	0.036	0.034	0.036	0.039	0.043	0.034	0.049	0.042	0.043	0.046	0.042	0.039	0.041
Dusadh	0.058	0.055	0.054	0.04	0.047	0.048	0.05	0.051	0.055	0.046	0.062	0.06	0.056	0.059	0.057	0.052	0.054
GIH	0.04	0.037	0.034	0.021	0.029	0.025	0.029	0.032	0.036	0.027	0.041	0.033	0.034	0.037	0.034	0.031	0.033
Kanjars	0.045	0.043	0.042	0.027	0.034	0.034	0.036	0.039	0.043	0.033	0.048	0.041	0.043	0.046	0.041	0.038	0.041
Kashmiri	0.031	0.026	0.024	0.01	0.017	0.015	0.017	0.022	0.025	0.015	0.031	0.023	0.025	0.029	0.025	0.02	0.023
Kol	0.046	0.044	0.044	0.028	0.036	0.035	0.038	0.04	0.043	0.035	0.05	0.043	0.044	0.046	0.043	0.04	0.042
Kshatriya	0.036	0.031	0.029	0.015	0.022	0.02	0.022	0.026	0.03	0.021	0.036	0.029	0.029	0.033	0.028	0.025	0.027
Lodi	0.043	0.04	0.04	0.025	0.031	0.031	0.033	0.036	0.038	0.031	0.046	0.041	0.041	0.045	0.041	0.037	0.039
Meghawal	0.038	0.035	0.033	0.018	0.026	0.023	0.027	0.03	0.033	0.024	0.039	0.032	0.034	0.037	0.034	0.031	0.033
Srivastava	0.037	0.031	0.033	0.015	0.023	0.024	0.02	0.029	0.031	0.02	0.042	0.035	0.036	0.04	0.033	0.031	0.033
Tharu	0.049	0.046	0.045	0.032	0.038	0.039	0.041	0.042	0.046	0.037	0.052	0.047	0.046	0.05	0.046	0.043	0.045
UP_Low_Caste	0.043	0.041	0.04	0.024	0.032	0.03	0.032	0.037	0.039	0.03	0.047	0.04	0.042	0.044	0.039	0.036	0.038
Vaish	0.035	0.03	0.03	0.016	0.022	0.021	0.022	0.027	0.03	0.02	0.036	0.031	0.031	0.035	0.03	0.026	0.029
Chenchu	0.076	0.071	0.071	0.057	0.064	0.067	0.069	0.067	0.072	0.064	0.079	0.077	0.073	0.076	0.075	0.07	0.071
Gond	0.046	0.045	0.044	0.029	0.035	0.037	0.035	0.04	0.043	0.034	0.052	0.045	0.047	0.051	0.044	0.041	0.044
Hakkipikki	0.054	0.051	0.052	0.036	0.042	0.045	0.044	0.048	0.05	0.041	0.058	0.053	0.054	0.058	0.053	0.05	0.052
Hallaki	0.046	0.045	0.045	0.029	0.036	0.035	0.037	0.041	0.043	0.036	0.051	0.041	0.045	0.048	0.044	0.041	0.045
Kamsali	0.047	0.046	0.046	0.029	0.035	0.037	0.037	0.041	0.044	0.035	0.051	0.046	0.047	0.051	0.048	0.043	0.047
Kurumba	0.048	0.047	0.046	0.032	0.038	0.036	0.04	0.043	0.046	0.037	0.052	0.045	0.047	0.05	0.047	0.044	0.047
Madiga	0.049	0.049	0.049	0.032	0.039	0.039	0.04	0.044	0.047	0.038	0.055	0.048	0.051	0.054	0.05	0.046	0.049
Naidu	0.044	0.041	0.042	0.025	0.033	0.032	0.033	0.038	0.039	0.031	0.048	0.041	0.043	0.045	0.043	0.039	0.042
North_Kannadi	0.057	0.055	0.055	0.039	0.046	0.046	0.048	0.05	0.054	0.046	0.061	0.055	0.056	0.059	0.056	0.052	0.055
Paniya	0.07	0.071	0.072	0.057	0.061	0.066	0.065	0.067	0.07	0.061	0.077	0.075	0.074	0.078	0.075	0.071	0.073
Piramalai_Kallars	0.049	0.048	0.047	0.033	0.039	0.039	0.04	0.044	0.047	0.038	0.054	0.047	0.048	0.052	0.049	0.045	0.048
Pulliyar	0.092	0.089	0.09	0.076	0.081	0.089	0.088	0.085	0.089	0.081	0.096	0.101	0.094	0.097	0.096	0.09	0.09
Sakilli	0.054	0.051	0.051	0.035	0.042	0.044	0.044	0.047	0.05	0.042	0.058	0.055	0.054	0.057	0.054	0.05	0.052
TN_Low_Caste	0.037	0.035	0.036	0.02	0.025	0.025	0.022	0.032	0.033	0.024	0.042	0.035	0.039	0.043	0.041	0.035	0.038
Velama	0.048	0.045	0.044	0.029	0.037	0.036	0.038	0.04	0.043	0.036	0.051	0.045	0.044	0.047	0.047	0.042	0.045
Vysya	0.052	0.049	0.048	0.033	0.04	0.04	0.042	0.044	0.048	0.038	0.056	0.052	0.05	0.053	0.052	0.047	0.05
Indian Jewish 1	0.044	0.037	0.038	0.026	0.03	0.033	0.031	0.034	0.036	0.028	0.043	0.039	0.038	0.042	0.044	0.036	0.039
IndianJewish 2	0.05	0.045	0.044	0.032	0.038	0.039	0.041	0.041	0.045	0.037	0.05	0.047	0.045	0.047	0.049	0.043	0.045
Indian Jewish 3	0.038	0.036	0.035	0.021	0.028	0.027	0.029	0.032	0.035	0.027	0.04	0.036	0.035	0.038	0.037	0.033	0.036
Indian Jewish 4	0.043	0.038	0.038	0.026	0.03	0.032	0.032	0.035	0.038	0.029	0.044	0.041	0.04	0.043	0.043	0.036	0.04
Munda	0.067	0.068	0.068	0.054	0.059	0.062	0.063	0.063	0.067	0.06	0.074	0.071	0.069	0.072	0.068	0.066	0.067
Aonaga	0.106	0.104	0.107	0.091	0.095	0.105	0.1	0.1	0.104	0.096	0.113	0.117	0.111	0.113	0.107	0.103	0.105
Garo	0.088	0.086	0.089	0.073	0.077	0.086	0.083	0.083	0.086	0.079	0.096	0.094	0.093	0.095	0.09	0.087	0.088
Naga	0.105	0.103	0.107	0.091	0.095	0.105	0.101	0.1	0.105	0.097	0.113	0.116	0.111	0.113	0.108	0.104	0.105
Nysha	0.102	0.099	0.101	0.086	0.09	0.099	0.096	0.095	0.1	0.091	0.108	0.11	0.106	0.108	0.104	0.099	0.1
Burmese	0.08	0.08	0.08	0.066	0.072	0.076	0.075	0.075	0.08	0.072	0.087	0.084	0.083	0.085	0.08	0.077	0.079
Cambodians	0.091	0.09	0.091	0.077	0.081	0.088	0.086	0.085	0.09	0.083	0.098	0.098	0.094	0.097	0.092	0.089	0.089
Dai	0.106	0.104	0.105	0.091	0.097	0.105	0.103	0.099	0.105	0.097	0.113	0.115	0.109	0.112	0.108	0.104	0.104
Daur	0.099	0.097	0.098	0.084	0.089	0.097	0.095	0.093	0.098	0.09	0.105	0.107	0.102	0.104	0.099	0.096	0.096
Han	0.11	0.107	0.108	0.095	0.1	0.107	0.106	0.103	0.109	0.101	0.116	0.115	0.112	0.114	0.11	0.106	0.107
Hezhen	0.101	0.099	0.101	0.087	0.092	0.1	0.098	0.095	0.1	0.093	0.109	0.11	0.105	0.107	0.102	0.099	0.099
Japanese	0.11	0.108	0.108	0.095	0.101	0.107	0.107	0.103	0.109	0.102	0.117	0.117	0.112	0.114	0.111	0.107	0.107
Lahu	0.115	0.111	0.112	0.098	0.103	0.114	0.111	0.107	0.112	0.104	0.121	0.126	0.117	0.12	0.116	0.112	0.112
Miaozu	0.107	0.105	0.106	0.092	0.097	0.106	0.104	0.1	0.105	0.098	0.114	0.116	0.11	0.112	0.108	0.105	0.104
Mongola	0.091	0.09	0.091	0.076	0.082	0.087	0.086	0.085	0.09	0.082	0.097	0.097	0.094	0.096	0.09	0.088	0.088
Naxi	0.104	0.102	0.104	0.09	0.095	0.103	0.101	0.098	0.104	0.096	0.112	0.114	0.108	0.111	0.106	0.102	0.103
Oroqens	0.101	0.099	0.1	0.086	0.092	0.099	0.097	0.095	0.1	0.092	0.108	0.109	0.105	0.107	0.101	0.098	0.098
She	0.113	0.109	0.111	0.098	0.102	0.112	0.109	0.105	0.112	0.104	0.12	0.123	0.116	0.119	0.114	0.111	0.11
Tu	0.09	0.088	0.09	0.075	0.08	0.087	0.085	0.084	0.089	0.081	0.096	0.095	0.093	0.095	0.09	0.087	0.087
Tujia	0.104	0.102	0.104	0.089	0.095	0.103	0.1	0.098	0.103	0.096	0.111	0.113	0.107	0.11	0.106	0.102	0.102
Xibo	0.092	0.09	0.091	0.076	0.082	0.088	0.087	0.086	0.091	0.083	0.099	0.098	0.094	0.096	0.091	0.088	0.089
Yizu	0.102	0.1	0.102	0.088	0.092	0.101	0.099	0.096	0.102	0.095	0.109	0.111	0.105	0.108	0.103	0.1	0.101

Colour key
Middle East
Jewish
Europe
Caucasus
Central Asia
South Asian
Southeast and East Asian

Supplementary Table. 3. The ANI (Ancestral North Indian) ancestry amond studied groups

Population	% ANI (SD)
**X= North India**	
GIH	47.78 (1.28)
Bhil	26.12 (1.89)
Indian Jewish 1	56.59 (2.27)
**X= South India**	
Kurumba	24.63 (1.77)
Sakilli	23.50 (2.22)
Malayan	8.45 (3.11)
Paniya	7.45 (2.46)
Indian Jewish 2	48.89 (1.84)
Indian Jewish 3	38.05 (1.65)
Indian Jewish 4	47.25 (2.29)

% ANI = Yoruba,French;X,S_Munda/Yoruba,French;Georgian,S_Munda

Supplementary Table. 4. The extended table showing formal test of Admixture using Alder

Admixed population	Surrogate population 1	Surrogate population 2	Time of Admixture (Generations)	SD	p value	Z score
Jewish 1	GIH	Yemenite Jewish	39.8	5.7	1.9 x 10-4	3.73
Jewish 1	GIH	Druze	37	8.6	6.9x10-5	3.98
Jewish 2	Kurumba	Yemenite Jewish	18.5	4.9	4.6 x 10-4	3.5
Jewish 2	Paniya	Yemenite Jewish	19	7.6	1.2 x 10-2	2.51
Jewish 2	Kurumba	Druze	16.3	4.7	5 x 10-4	3.48
Jewish 2	Paniya	Druze	17.2	9.8	7.8 x 10-2	1.76
Jewish 3	Kurumba	Yemenite Jewish	131.7	122.9	0.61	0.5
Jewish 3	Paniya	Yemenite Jewish	82	41.7	4.9 x 10-2	1.97
Jewish 3	Kurumba	Druze	42.9	76.7	0.48	0.63
Jewish 3	Paniya	Druze	27.4	7.6	8.4 x 10-4	3.34
Jewish 3	Kurumba	-	36.5	11.3	1.6x 10-4	3.23
Jewish 4	Kurumba	Yemenite Jewish	30.8	21.1	0.14	1.46
Jewish 4	Paniya	Yemenite Jewish	10.1	5.2	5.2 x 10-2	1.94
Jewish 4	Kurumba	Druze	53.1	11.3	3.1x10-5	4.17
Jewish 4	Paniya	Druze	14.8	39.1	0.7	0.38

Supp. Table 5. The mtDNA conding and control region mutation of Indian Jewish and their neighbouring Indian populations

ID	Group	Code	HVSI (-16000)	HVSII and Coding	Haplogroup	Reference
DMB00313	Mumbai Jewish	Jewish 1	256-352	146-260	H14	Behar et al. 2008
DMB00321	Mumbai Jewish	Jewish 1	519	183-263	H13a2a1	Behar et al. 2008
DMB00330	Mumbai Jewish	Jewish 1	519	183-263	H13a2a1	Behar et al. 2008
DMB00316	Mumbai Jewish	Jewish 1	051-223-304	56d-58a-64.1T-66t-73-153-263	M39	Behar et al. 2008
DMB00311	Mumbai Jewish	Jewish 1	093-223-304	56d-73-153-263	M39	Behar et al. 2008
DMB00305	Mumbai Jewish	Jewish 1	093-223-304	56d-73-153-263	M39	Behar et al. 2008
DMB00306	Mumbai Jewish	Jewish 1	129-183-223-278-519	73-93-146-195-263	M	Behar et al. 2008
DMB00317	Mumbai Jewish	Jewish 1	129-223-362-519	73-263	M	Behar et al. 2008
DMB00310	Mumbai Jewish	Jewish 1	192-223-519	73-199-263	M	Behar et al. 2008
DMB00322	Mumbai Jewish	Jewish 1	223-263-519-527	73-152-207-263-15355-15968	M64	Behar et al. 2008
DMB00314	Mumbai Jewish	Jewish 1	223-301-519	73-146	M	Behar et al. 2008
DMB00331	Mumbai Jewish	Jewish 1	223-301-519	73-146-263	M	Behar et al. 2008
DMB00327	Mumbai Jewish	Jewish 1	223-304	56d-58a-65.1T-73-153-263	M39a1	Behar et al. 2008
DMB00315	Mumbai Jewish	Jewish 1	069-166d-223-519	73-146-195a-263	M30c1a1	Behar et al. 2008
DMB00328	Mumbai Jewish	Jewish 1	069-166d-223-519	73-146-195a-263	M30c1a1	Behar et al. 2008
DMB00334	Mumbai Jewish	Jewish 1	069-166d-223-519	73-146-195a-263	M30c1a1	Behar et al. 2008
DMB00336	Mumbai Jewish	Jewish 1	069-166d-223-519	73-146-195a-263	M30c1a1	Behar et al. 2008
DMB00425	Mumbai Jewish	Jewish 1	166-223-271-311-519	56d-58a-65.1T-73-152-263	M39a1	Behar et al. 2008
DMB00319	Mumbai Jewish	Jewish 1	166-223-311-519	56d-58a-65.1T-73-146-152-207-263	M39a1	Behar et al. 2008
DMB00320	Mumbai Jewish	Jewish 1	166-223-311-519	56d-58a-65.1T-73-146-152-207-263	M39a1	Behar et al. 2008
DMB00329	Mumbai Jewish	Jewish 1	166-223-311-519	56d-58a-65.1T-73-152-189-207-263	M39a1	Behar et al. 2008
DMB00309	Mumbai Jewish	Jewish 1	166-223-311-519	56d-58- 65.1T-73-152-207-263	M39a1	Behar et al. 2008
DMB00323	Mumbai Jewish	Jewish 1	166-223-311-519	56d-58- 65.1T-73-152-207-263	M39a1	Behar et al. 2008
DMB00324	Mumbai Jewish	Jewish 1	166-223-311-519	56d-58- 65.1T-73-152-207-263	M39a1	Behar et al. 2008
DMB00325	Mumbai Jewish	Jewish 1	166-223-311-519	56d-58- 65.1T-73-152-207-263	M39a1	Behar et al. 2008
DMB00326	Mumbai Jewish	Jewish 1	166-223-311-519	56d-58- 65.1T-73-152-207-263	M39a1	Behar et al. 2008
DMB00332	Mumbai Jewish	Jewish 1	166-223-311-519	56d-58- 65.1T-73-152-207-263	M39a1	Behar et al. 2008
DMB00333	Mumbai Jewish	Jewish 1	166-223-311-519	56d-58- 65.1T-73-152-207-263	M39a1	Behar et al. 2008
DMB00337	Mumbai Jewish	Jewish 1	166-223-311-519	56d-58- 65.1T-73-152-207-263	M39a1	Behar et al. 2008
DMB00335	Mumbai Jewish	Jewish 1	166-223-311-519	56d-58- 65.1T-73-152-207-263	M39a1	Behar et al. 2008
DMB00307	Mumbai Jewish	Jewish 1	166-223-311-519	56d-58- 65.1T-73-152-207-263	M39a1	Behar et al. 2008
DMB00308	Mumbai Jewish	Jewish 1	292-497-519	73-263-373	R30b	Behar et al. 2008
DMB00312	Mumbai Jewish	Jewish 1	292-497-519	73-263-373	R30b	Behar et al. 2008
DMB00318	Mumbai Jewish	Jewish 1	292-497-519	73-263-373	R30b	Behar et al. 2008
DMB01105	Cochin Jewish	Jewish 4	172-182-183-189-218-223-362-399	73-150-263	D5	Behar et al. 2008
DMB01118	Cochin Jewish	Jewish 4	126-223	56d-58-73-153-234-263	M	Behar et al. 2008
DMB01102	Cochin Jewish	Jewish 4	129-223-234-519	73-263	M	Behar et al. 2008
DMB01116	Cochin Jewish	Jewish 4	189-223-293-519	73-195a-263	M30	Behar et al. 2008
DMB01084	Cochin Jewish	Jewish 4	223-263-519-527	73-152-207-263-15355-15968	M64	Behar et al. 2008
DMB01089	Cochin Jewish	Jewish 4	223-263-519-527	73-152-207-263-15355-15968	M64	Behar et al. 2008
DMB01081	Cochin Jewish	Jewish 4	223-263-519-527	73-152-207-263-15355-15968	M64	Behar et al. 2008
DMB01082	Cochin Jewish	Jewish 4	223-263-519-527	73-152-207-263-15355-15968	M64	Behar et al. 2008
DMB01088	Cochin Jewish	Jewish 4	223-263-519-527	73-152-207-263-15355-15968	M64	Behar et al. 2008
DMB01114	Cochin Jewish	Jewish 4	223-263-519-527	73-152-207-263-15355-15968	M64	Behar et al. 2008
DMB01117	Cochin Jewish	Jewish 4	223-263-519-527	73-152-207-263-15355-15968	M64	Behar et al. 2008
DMB01121	Cochin Jewish	Jewish 4	223-263-519-527	73-152-207-263-15355-15968	M64	Behar et al. 2008
DMB01092	Cochin Jewish	Jewish 4	223-257-519	73-263	M5a1	Behar et al. 2008
DMB01096	Cochin Jewish	Jewish 4	223-257-519	73-263	M5a1	Behar et al. 2008
DMB01097	Cochin Jewish	Jewish 4	223-257-519	73-263	M5a1	Behar et al. 2008
DMB01099	Cochin Jewish	Jewish 4	223-257-519	10398-10400-12477-12681-15043-15301	M5a1	Behar et al. 2008
DMB01100	Cochin Jewish	Jewish 4	223-257-519	10398-10400-12477-12681-15043-15301	M5a1	Behar et al. 2008
DMB01108	Cochin Jewish	Jewish 4	223-257-519	10398-10400-12477-12681-15043-15301	M5a1	Behar et al. 2008
DMB01109	Cochin Jewish	Jewish 4	223-257-519	10398-10400-12477-12681-15043-15301	M5a1	Behar et al. 2008
DMB01111	Cochin Jewish	Jewish 4	223-257-519	10398-10400-12477-12681-15043-15301	M5a1	Behar et al. 2008
DMB01115	Cochin Jewish	Jewish 4	223-257-519	10398-10400-12477-12681-15043-15301	M5a1	Behar et al. 2008
DMB01119	Cochin Jewish	Jewish 4	223-257-519	10398-10400-12477-12681-15043-15301	M5a1	Behar et al. 2008
DMB01120	Cochin Jewish	Jewish 4	223-257-519	10398-10400-12477-12681-15043-15301	M5a1	Behar et al. 2008
DMB01123	Cochin Jewish	Jewish 4	223-257-519	10398-10400-12477-12681-15043-15301	M5a1	Behar et al. 2008
DMB01083	Cochin Jewish	Jewish 4	223-356	73-199-204-263	N1a	Behar et al. 2008
DMB01125	Cochin Jewish	Jewish 4	223-356	73-199-204-263	N1a	Behar et al. 2008
DMB01085	Cochin Jewish	Jewish 4	172-278-519	73-263	R30a	Behar et al. 2008
DMB01087	Cochin Jewish	Jewish 4	172-278-519	73-263	R30a	Behar et al. 2008
DMB01113	Cochin Jewish	Jewish 4	172-278-519	73-263	R30a	Behar et al. 2008
DMB01122	Cochin Jewish	Jewish 4	189-304-519-524-526	73-93-200-263	R5	Behar et al. 2008
DMB01090	Cochin Jewish	Jewish 4	rCRS	73-152-263	R	Behar et al. 2008
DMB01094	Cochin Jewish	Jewish 4	rCRS	73-263	R	Behar et al. 2008
DMB01106	Cochin Jewish	Jewish 4	rCRS	73-263	R	Behar et al. 2008
DMB01093	Cochin Jewish	Jewish 4	266-304-311-519-524	73-93-200-263	R5a1a	Behar et al. 2008
DMB01103	Cochin Jewish	Jewish 4	266-304-311-519-524	73-93-200-263	R5a1a	Behar et al. 2008
DMB01104	Cochin Jewish	Jewish 4	266-304-311-519-524	73-93-200-263	R5a1a	Behar et al. 2008
DMB01107	Cochin Jewish	Jewish 4	266-304-311-519-524	73-93-200-263	R5a1a	Behar et al. 2008
DMB01124	Cochin Jewish	Jewish 4	266-304-311-519-524	73-93-200-263	R5a1a	Behar et al. 2008
DMB01095	Cochin Jewish	Jewish 4	093-129-183c-189-519	73-195-263	U1a	Behar et al. 2008
DMB01086	Cochin Jewish	Jewish 4	093-129-189-222-249	73-152-263-285	U1	Behar et al. 2008
DMB01091	Cochin Jewish	Jewish 4	093-129-189-222-249	73-152-263-285	U1	Behar et al. 2008
DMB01098	Cochin Jewish	Jewish 4	093-129-189-222-249	73-152-263-285	U1	Behar et al. 2008
DMB01101	Cochin Jewish	Jewish 4	093-129-189-222-249	73-152-263-285	U1	Behar et al. 2008
DMB01110	Cochin Jewish	Jewish 4	093-129-189-222-249	73-152-263-285	U1	Behar et al. 2008
DMB01112	Cochin Jewish	Jewish 4	292-497-519	73-152-263-12414	W	Behar et al. 2008
B86	Cochin Jewish	Jewish 3	129-172-234-266-274-309-362	12285-12705	R6a1	Present Study
B87	Cochin Jewish	Jewish 3	257-266-304-309-325-335-356	73-152-189-263-489-522d-523d-12705-15385	R5a2b	Present Study
B102	Cochin Jewish	Jewish 3	051-209-239-292-352-353	73-195-263-459-12106-12308-12372-12705	U2b2	Present Study
B101	Cochin Jewish	Jewish 3	223-304	153-485-489-10398-10400-14985-12705-15043-15301-15938	M39b	Present Study
B106	Cochin Jewish	Jewish 3	224	73-263-4971-2308-12372-12705-15355	K1a4d	Present Study
D1	Cochin Jewish	Jewish 3	040-295-311	480-10142-12207-12705	R30b	Present Study
D10	Cochin Jewish	Jewish 3	168-172-188-239	12705	R	Present Study
D11	Cochin Jewish	Jewish 3	320-356	195-499-12308-12372-12705-15693	U4	Present Study
D3	Cochin Jewish	Jewish 3	223	10398-10400-10556-12007-15043-15301	M37	Present Study
D4	Cochin Jewish	Jewish 3	051-234	12172-12308-12372-12705-15214	U2c	Present Study
D5	Cochin Jewish	Jewish 3	189-249	12308-372-705-15217	U1	Present Study
D6	Cochin Jewish	Jewish 3	154-354	12127-12705	R8	Present Study
D7	Cochin Jewish	Jewish 3	189-240-324-402	1438-12127-12705	R8	Present Study
D8	Cochin Jewish	Jewish 3	189-270	12308-12372-12618-12705	U5b1b	Present Study
D12	Cochin Jewish	Jewish 3	153-485-489-15938-223-304	153-485-489-10398-10400-15043-15301	M39b	Present Study
E5	Cochin Jewish	Jewish 3	223-287-304	146-489-10398-10400-15043-15301	M	Present Study
E6	Cochin Jewish	Jewish 3	292-362	196-198-204-207-228-234-15884	R31b	Present Study
E7	Cochin Jewish	Jewish 3	126-154-223-354	482-489-523del-10398-10400-12467-15043-15301	M3b	Present Study
E8	Cochin Jewish	Jewish 3	354	195-1438-12127-12705	R8	Present Study
E9	Cochin Jewish	Jewish 3	126-223	150-309+c-482-489-10398-10400-15043-15301	M3	Present Study
F10	Cochin Jewish	Jewish 3	184-223-260-298	152-489-10398-10400-12241del-15043-15261-15301-15326-15486	Z1	Present Study
F11	Cochin Jewish	Jewish 3	129-172-234-266-274-309-362	459-12285-12705-14990-15326-15385	R6a1a	Present Study
F12	Cochin Jewish	Jewish 3	067-182-183-327A	12705-15218	HV1a3	Present Study
F13	Cochin Jewish	Jewish 3	067-182-183-327A	12705-15218	HV1a3	Present Study
F14	Cochin Jewish	Jewish 3	172-192	12133-12285-12705	R6a2	Present Study
F15	Cochin Jewish	Jewish 3	223-257	10398-10400-12477-12681-15043-15301	M5a	Present Study
F16	Cochin Jewish	Jewish 3	067-182-183-327A	12705-15218	HV1a3	Present Study

F17	Cochin Jewish	Jewish 3	rCRS	12705-15218	HV1a3	Present Study
1	Cochin Jewish	Jewish 3	129-291	489-10398-10400-12477-15043-15287-15301-15379	M5a1	Present Study
10	Cochin Jewish	Jewish 3	309-318T		U7	Present Study
12	Cochin Jewish	Jewish 3	266-274-362	459-12285-12705	R6a	Present Study
13	Cochin Jewish	Jewish 3	040-311	480-10142-12207-12705	R30b	Present Study
14	Cochin Jewish	Jewish 3	042-223-274-319-320-362	10398-10400-15043-15253-15301-15670	M2a1a	Present Study
15	Cochin Jewish	Jewish 3	223	10398-10400-12561-15043-15301-15924	M35a	Present Study
16	Cochin Jewish	Jewish 3	126-185-223-519	73-195-263-482-489-10398-10400-15018-15043-15301	M3	Present Study
17	Cochin Jewish	Jewish 3	182C-183C-189-249	10253-12308-12372-12705-15217-15954C	U1	Present Study
19	Cochin Jewish	Jewish 3	093-223	10398-10400-15043-15301-15924	M35a	Present Study
2	Cochin Jewish	Jewish 3	093-129	12705	R	Present Study
20	Cochin Jewish	Jewish 3	257-266-304-309-325-335-356	12705-15385	R5a2b	Present Study
22	Cochin Jewish	Jewish 3	111-187-223-274-319-320-362	10398-10400-12172-15043-15253-15301-15670	M2a1a	Present Study
23	Cochin Jewish	Jewish 3	111-187-223-274-319-320-362	10398-10400-12172-15043-15253-15301-15670	M2a1a	Present Study
24	Cochin Jewish	Jewish 3	093-129-362	12285-12705	R6a	Present Study
26	Cochin Jewish	Jewish 3	140-183C-189-266A	73-195-263-4829bp del-10398-12705-15235	B5a	Present Study
25	Cochin Jewish	Jewish 3	111-187-223-274-319-320-362	10398-10400-12172-15043-15253-15301-15670	M2a1a	Present Study
27	Cochin Jewish	Jewish 3	129-172-234-266-274-309-362	12285-12705-14990-15326-15385	R6a1a	Present Study
28	Cochin Jewish	Jewish 3	111-184-185-186-189-223-295	10398-10400-10556-12007-15043-15301	M37e	Present Study
29	Cochin Jewish	Jewish 3	223	10398-10400-12007-15043-15301-15431	M30	Present Study
3	Cochin Jewish	Jewish 3	126-185-223	73-195-263-482-489-10398-10400-15018-15043-15301	M3	Present Study
30	Cochin Jewish	Jewish 3	257-266-304-309-325-335-356	12705-15385	R5a2b	Present Study
31	Cochin Jewish	Jewish 3	951-126-185-223	73-195-263-482-489-10398-10400-15018-15043-15301	M3	Present Study
32	Cochin Jewish	Jewish 3	129-223-291	10398-10400-12477-15043-15287-15301-15379	M5a1	Present Study
33	Cochin Jewish	Jewish 3	223	10398-10400-12007-15043-15301-15431	M30	Present Study
34	Cochin Jewish	Jewish 3	092-111-187-189-223-274-319-320-362	10398-10400-12172-15043-15253-15301-15670	M2a1a1a1	Present Study
35	Cochin Jewish	Jewish 3	167-172-278	12705-12714	R30a	Present Study
4	Cochin Jewish	Jewish 3	093-176-223-362	152-195A-48910398-10400-12007-15043-15301-15314-15431	M30e	Present Study
40	Cochin Jewish	Jewish 3	167-172-176-278	12705-12714	R30a	Present Study
41	Cochin Jewish	Jewish 3	257-266-304-309-325-335-356	12705-15385	R5a2b	Present Study
42	Cochin Jewish	Jewish 3	182C-183C-189-249	10253-12308-12372-12705-15217-15954C	U1	Present Study
43	Cochin Jewish	Jewish 3	172-278	12705-12714	R30a	Present Study
44	Cochin Jewish	Jewish 3	223	10398-10400-12007-15043-15301-15431	M30	Present Study
45	Cochin Jewish	Jewish 3	167-172-278-318T	12308-12372-12705	U7	Present Study
46	Cochin Jewish	Jewish 3	266-289	10398-10400-12561-15043-15301-15924	M35a	Present Study
47	Cochin Jewish	Jewish 3	304-309-325-356	12705-15226-15385	R5a2b	Present Study
48	Cochin Jewish	Jewish 3	223-266-289	10398-10400-12007-12618-15043-15301-15924	M	Present Study
49	Cochin Jewish	Jewish 3	223-399	10398-10400-12007-15043-15301-15314-15431-15111	M30e	Present Study
5	Cochin Jewish	Jewish 3	051-129C-183C-189-362	152-204-217-508-12308-12372-12557-12705-15018	U2e	Present Study
6	Cochin Jewish	Jewish 3	051-129C-183C-189-362	152-204-217-508-12308-12372-12557-12705-15018	U2e	Present Study
9	Cochin Jewish	Jewish 3	218-309-318C	12308-12372-12705	U7	Present Study
E1	Cochin Jewish	Jewish 3	129-223	12705-15218-15927	HV1a1	Present Study
7	Cochin Jewish	Jewish 3	184-223-260-298	10398-10400-12241del-15043-15261-15301-15326-15486	Z1	Present Study
D5	Cochin Jewish	Jewish 3	182C-183C-189-249	285-10253-12308-12372-12705-15217-15954C	U1	Present Study
D9	Cochin Jewish	Jewish 3	040-295-311	480-10142-12207-12705	R30b	Present Study
37	Cochin Jewish	Jewish 3	126-185-223	73-195-263-482-489-10398-10400-15018-15043-15301	M3	Present Study
50	Cochin Jewish	Jewish 3	126-185-223	73-195-263-482-489-10398-10400-15018-15043-15301	M3	Present Study
E3	Cochin Jewish	Jewish 3	126-223	150-309+c-482-489-10398-10400-15043-15301	M3	Present Study
E4	Cochin Jewish	Jewish 3	111-223-311-357	10398-10400-15043-15301	M4	Present Study
8	Cochin Jewish	Jewish 3	218-309-318C	12308-12372-12705	U7	Present Study
10	Cochin Jewish	Jewish 3	218-309-318C	12308-12372-12705	U7	Present Study
E2	Cochin Jewish	Jewish 3	189-192-270	12308-12372-12618-12705	U5b1b	Present Study
18	Cochin Jewish	Jewish 3	051-129C-183C-189-362	152-204-217-508-12308-12372-12557-12705-15018	U2e	Present Study
24	Cochin Jewish	Jewish 3	129-172-234-266-274-309-362	12285-12705-14990-15326-15385	R6a1a	Present Study
25	Cochin Jewish	Jewish 3	051-206C-230-304-311-519	12308-12372-12705-15049-15940	U2a1	Present Study
36	Cochin Jewish	Jewish 3	126-185-223-519	73-195-263-482-489-10398-10400-15018-15043-15301	M3	Present Study
29	Cochin Jewish	Jewish 3	182C-183C-189-249	10253-12308-12372-12705-15217	U1	Present Study
19	Cochin Jewish	Jewish 3	257-266-304-309-325-335-356	12705-15385	R5a2b	Present Study
21	Cochin Jewish	Jewish 3	069-274-362	10398-10400-12007-15043-15301-15431	M30	Present Study
H25	Cochin Jewish	Jewish 3	111-144-223-224-311	9545	N5b	Present Study
Z1	Cochin Jewish	Jewish 3	184-223-260-298	10398-10400-12285-15043-15130-15253-15301	Z	Present Study
Z2	Cochin Jewish	Jewish 3	224	73-263-497-12308-12372-12705-15355	K1a4d	Present Study
Z3	Cochin Jewish	Jewish 3	184-223-260-298	10398-10400-12285-15043-15130-15253-15301	Z	Present Study
Z4	Cochin Jewish	Jewish 3	224	73-263-497-12308-12372-12705-15355	K1a4d	Present Study
83	Cochin Jewish	Jewish 3	274-301	199-204-10398-12705-13437-15226-15301	N1a2	Present Study
84	Cochin Jewish	Jewish 3	196-203-223	10398-10400	M	Present Study
D21	Cochin Jewish	Jewish 3	129-519	10398-10400-12477-15043-15301	M5a	Present Study
D25	Cochin Jewish	Jewish 3	129-519	10398-10400-12477-15043-15301	M5a	Present Study
HV 32	Cochin Jewish	Jewish 3	129-265C-311	10398-10400-12172-12477	M5	Present Study
KH83	Cochin Jewish	Jewish 3	093-126-290	10398-10400-15043-15301-15924	M35a	Present Study
K001	Cochin Jewish	Jewish 3	224-234-311	10550-12308-12372-12705-15924	K1a1b1a	Present Study
NN94	Cochin Jewish	Jewish 3	304	10398-10400-15043-15301-15928	M35b	Present Study
RJ76	Cochin Jewish	Jewish 3	234-249	285-12308-12372-12705	U1	Present Study
21	Cochin Jewish	Jewish 3	126-311	482-10398-10400-15043-15301	M3	Present Study
D126	Cochin Jewish	Jewish 3	239-298-327-357	10398-10400-15043-15301	C4a4a	Present Study
2820	Cochin Jewish	Jewish 3	224-234-311	10550-12308-12372-12705-15924	K1a1b1a	Present Study
4335	Cochin Jewish	Jewish 3	129	10398-10400-12477-15043-15301	M5a	Present Study
4355	Cochin Jewish	Jewish 3	129	10398-10400-12477-12705-15043-15301	M5a	Present Study
4359	Cochin Jewish	Jewish 3	129	10398-10400-12477-15043-15301	M5a	Present Study
5948	Cochin Jewish	Jewish 3	224-234-311	10550-12308-12372-12705-15924	K1a1b1a	Present Study
13	Cochin Jewish	Jewish 3	126-311	482-10398-10400-15043-15301	M3	Present Study
4360	Cochin Jewish	Jewish 3	129	10398-10400-12477-12705-15043-15301	M5a	Present Study
32H	Cochin Jewish	Jewish 3	129-172-234	10398-10400	M5	Present Study
5923	Cochin Jewish	Jewish 3	129	10398-10400-12477-15043-15301-15909	M5a	Present Study
D35	Cochin Jewish	Jewish 3	129	10398-10400-12477-15043-15301-15909	M5a	Present Study
4366	Cochin Jewish	Jewish 3	172-183C-189-274-319-320	10398-10400-15043-15253-15301-15670	M2a1a1a1	Present Study
4367	Cochin Jewish	Jewish 3	172-183C-189-274-319-320	10398-10400-15043-15253-15301-15670	M2a1a1a1	Present Study
4362	Cochin Jewish	Jewish 3	172-183C-189-274-319-320	10398-10400-15043-15253-15301-15670	M2a1a1a1	Present Study
V07	Cochin Jewish	Jewish 3	145-176-234-261-311	10398-10400-15043-15301	M4a	Present Study
D88	Cochin Jewish	Jewish 3	129	10398-10400-12477-15043-15301	M5a	Present Study
S90	Cochin Jewish	Jewish 3	129-265C-390	10398-10400-12172-12477	M5	Present Study
53	Cochin Jewish	Jewish 3	223-318T	10398-10400-12498	M18	Present Study
6021	Cochin Jewish	Jewish 3	172-234-270-274-319-352	10398-10400-15043-15253-15301-15670	M2a1a1a1	Present Study
K004	Cochin Jewish	Jewish 3	172-234-270-274-319-352	10398-10400-15043-15253-15301-15670	M2a1a1a1	Present Study
38	Cochin Jewish	Jewish 3	224-234	10398-10550-12308-12372-12705	K1a1b1a	Present Study
D5	Cochin Jewish	Jewish 3	183C-189-311	12133-12705	R6a2	Present Study
4338	Cochin Jewish	Jewish 3	129	10398-10400-12477-15043-15301	M5a	Present Study
R17	Cochin Jewish	Jewish 3	311	12133-12705	R6a2	Present Study
5923	Cochin Jewish	Jewish 3	129-223-519	10398-10400-12477-15043-15301-15909	M5a	Present Study
D119	Cochin Jewish	Jewish 3	129-223-519	10398-10400-12477-15043-15287-15301-15379	M5a1b	Present Study
4344	Cochin Jewish	Jewish 3	129-223	263-489-734-10398-10400-12477-15043-15287-15301-15379	M5a2	Present Study
B67	Cochin Jewish	Jewish 3	051-129C-183C-189-362	152-204-217-508-12308-12372-12557-12705-15018	U2e	Present Study
B69	Cochin Jewish	Jewish 3	051-092-129C-183C-189-362	152-204-217-508-12308-12372-12557-12705-15018	U2e	Present Study
B52	Cochin Jewish	Jewish 3	223-368	73-152-195A-263-489-10398-10400-15043-15301	M30e	Present Study
B55	Cochin Jewish	Jewish 3	187-189-207-309-318T	12308-12372-12557-12705	U7	Present Study
B64	Cochin Jewish	Jewish 3	187-189-207-309-318T	12308-31272-12557-12705	U7	Present Study
B63	Cochin Jewish	Jewish 3	051-234-247-254-311	73-263-12308-12372-12557-12705	U2c	Present Study
B68	Cochin Jewish	Jewish 3	182C-183C-189-249	10253-12308-12372-12705-15217	U1	Present Study
B76	Cochin Jewish	Jewish 3	223-311	10398-10400-12007-12705-15043-15301	M4	Present Study

B77	Cochin Jewish	Jewish 3	217-325	12133-12285-12705	R6a2	Present Study
B56	Cochin Jewish	Jewish 3	189-224-311	73-146-150-263-12308-12372-12705	K	Present Study
B57	Cochin Jewish	Jewish 3	186-189-223-270-319-352	10398-10400-15043-15301-15670	M2a1	Present Study
B74	Cochin Jewish	Jewish 3	266-304-309-325-356	12705-15385	R5a2b	Present Study
B76	Cochin Jewish	Jewish 3	266-318-320-362	73-195-263-459-12285-12705-14990-15326-15385	R6a1a	Present Study
B84	Cochin Jewish	Jewish 3	147-189-217-235	9bp del-5108	B4c2	Present Study
B51	Cochin Jewish	Jewish 3	223-311	73-263-482-489-523d-535d-10398-10400-15043-15301	M3	Present Study
B53	Cochin Jewish	Jewish 3	223-311	10398-10400-12007-15043-15301-15431	M30	Present Study
B73	Cochin Jewish	Jewish 3	266-304-309-325-356	12705-15385	R5a2b	Present Study
B54	Cochin Jewish	Jewish 3	rCRS	12133-12705	R6a2	Present Study
H36	Cochin Jewish	Jewish 3	183C-189-266A-362	9bp del-10398-12705-15235	B5a	Present Study
B103	Cochin Jewish	Jewish 3	126-223-309	73-146-204-263-482-489-10084-10398-10400-15043-15301	M3	Present Study
B104	Cochin Jewish	Jewish 3	129-172-234-266-274-309-362	12285-12705-14990-15326-15385	R6a1a	Present Study
B105	Cochin Jewish	Jewish 3	093-223-309	73-199-263-482-489-10398-10400-15043-15301	M35a	Present Study
B106"	Cochin Jewish	Jewish 3	223-309-318T	73-146-263-523-24d-12308-12372-12705	U7	Present Study
B3	Cochin Jewish	Jewish 3	176-223-270-274-319-352	73-204-263-447G-489-709-10398-10400-15018-15043-15301-15670	M2a1a1a1	Present Study
B4	Cochin Jewish	Jewish 3	176-291-352-353	12106-12308-12372-12705-15049	U2b2	Present Study
B42	Cochin Jewish	Jewish 3	126-185-223-309	73-195-263-482-489-10398-10400-15018-15043-15301	M3	Present Study
B43	Cochin Jewish	Jewish 3	223-234-362	73-195A-263-523d-525d-10398-10400-12007-15043-15301-15431	M30	Present Study
B52	Cochin Jewish	Jewish 3	051-092-129C-183C-189-362	152-204-217-508-12308-12372-12557-12705-15018	U2e	Present Study
B65	Cochin Jewish	Jewish 3	223-320	73-263-482-489-10398-10400-15043-15301	M3	Present Study
B66	Cochin Jewish	Jewish 3	051-223-309	73-263-482-489-10398-10400-15043-15301	M35a	Present Study
B72	Cochin Jewish	Jewish 3	111-187-223-274-319-320-362	73-143-195-263-337-447G-489-10398-10400-12561-15018-15043-15130-15253-15301-15670	M2b	Present Study
B75	Cochin Jewish	Jewish 3	129-172-234-266-274-309-362	73-195-263-459-12285-12705-15326-15385	R6a1a	Present Study
B85	Cochin Jewish	Jewish 3	111-187-223-274-319-320-362	73-143-195-263-337-447G-489-10398-10400-12172-15705-14985-15018-15043-15130-15253-15301-15670	M2b	Present Study
B86	Cochin Jewish	Jewish 3	129-172-234-274-309-362	73-146-195-263-459-12285-12705-14990-15326-15385	R6a1a	Present Study
B87B	Cochin Jewish	Jewish 3	189-192-270	12308-12372-12618-12705	U5b1b	Present Study
C17	Cochin Jewish	Jewish 3	257-304-309-335-356	12705-15226-15385	R5a2b	Present Study
C17B	Cochin Jewish	Jewish 3	257-304-309-335-356	73-152-189-215-523d-525d-12705-15385	R5a2b	Present Study
C36	Cochin Jewish	Jewish 3	309-318T		U7	Present Study
C20	Cochin Jewish	Jewish 3	257-304-309-335-356	12705-15385	R5a2b	Present Study
H32	Cochin Jewish	Jewish 3	129-172-234-274-309-362	12285-12705-14990-15326-15385	R6a1a	Present Study
K1	Cochin Jewish	Jewish 3	051-209-239-292-352-353	12106-12308-12372-12705	U2b2	Present Study
K2	Cochin Jewish	Jewish 3	183C-189-304	249d-10310-10609-12705	F1	Present Study
K3	Cochin Jewish	Jewish 3	051-209-239-292-352-353	12106-12308-12372-12705	U2b2	Present Study
K4	Cochin Jewish	Jewish 3	126-223	482-10398-10400-15043-15301	M3	Present Study
K5	Cochin Jewish	Jewish 3	181-309-318T	12172-12308-12372	U7	Present Study
B71	Cochin Jewish	Jewish 3	051-092-129C-183C-189-362	152-204-217-508-12308-12372-12557-12705-15018	U2e	Present Study
B61	Cochin Jewish	Jewish 3	051-092-129C-183C-189-362	152-204-217-508-12308-12372-12557-12705-15018	U2e	Present Study
C21	Cochin Jewish	Jewish 3	rCRS	12133-12705	R6a2	Present Study
B51	Cochin Jewish	Jewish 3	223	73-263-489-523d-525d10398-10400-12007-15043-15301-15431	M30	Present Study
B56	Cochin Jewish	Jewish 3	185-223-260-298	73-146-150-263-482-489-10398-10400-12241del-15043-15261-15301-15326-15486	Z1	Present Study
B67	Cochin Jewish	Jewish 3	126-223	482-10398-10400-15043-15301	M3	Present Study
B70	Cochin Jewish	Jewish 3	126-223	482-10398-10400-15043-15301	M3	Present Study
B79	Cochin Jewish	Jewish 3	129-172-234-266-274-309-362	73-263-459-12285-12705-14990-15326-15385	R6a1a	Present Study
C1	Cochin Jewish	Jewish 3	223	73-195A-263-523d-525d-725-10398-10400-12007-15043-15301-15431	M30	Present Study
C2	Cochin Jewish	Jewish 3	111-187-223-274-319-320-362	73-143-195-263-337-447G-489-10398-10400-12172-15705-15018-15043-15130-15253-15301-15670	M2b	Present Study
C3	Cochin Jewish	Jewish 3	104-129-223	10398-10400-12477-15043-15301	M5a	Present Study
C4	Cochin Jewish	Jewish 3	111-187-223-274-319-320-362	73-143-195-263-337-447G10398-10400-12172-15705-15043-15130-15253-15301-15670	M2b	Present Study
C5	Cochin Jewish	Jewish 3	129-172-234-266-274-309-362	12285-12705-14990-15326-15385	R6a1a	Present Study
C6	Cochin Jewish	Jewish 3	126-185-223-519	73-195-263-482-489-10398-10400-15018-15043-15301	M3	Present Study
C7	Cochin Jewish	Jewish 3	093-129-231-356-362	10398-10400-15043-15301	M6a1	Present Study
C8	Cochin Jewish	Jewish 3	951-126-185-223-519	73-195-263-482-489-10398-10400-15018-15043-15301	M3	Present Study
C9	Cochin Jewish	Jewish 3	69	12007-12705-15235	J1c2a	Present Study
C10	Cochin Jewish	Jewish 3	185-223-260-298	73-152-26310398-10400-12241del-15043-15261-15301-15326-15486	Z1	Present Study
C11	Cochin Jewish	Jewish 3	051-187-189-206C-230	12308-12357-12372-12705-15049	U2a1	Present Study
C12	Cochin Jewish	Jewish 3	051-092-129C-183C-189C-362	152-204-217-508-12308-12372-12557-12705-15018	U2e	Present Study
C13	Cochin Jewish	Jewish 3	017-126-145-223	10398-10400-15440	M31a2	Present Study
C15	Cochin Jewish	Jewish 3	189-249-311-327	12308-12357-12372-12705	U1	Present Study
C14	Cochin Jewish	Jewish 3	051-206C-230-304-311-519	12308-12357-12372-12705-15049-15940	U2a1	Present Study
C16	Cochin Jewish	Jewish 3	051-206C-230-304-311-519	73-195A-263-523d-525d-70912308-12357-12372-12705-15049-15940	U2a1	Present Study
C17	Cochin Jewish	Jewish 3	111-223-274	73-152-189-215-522-524d-12285-12705	R6a	Present Study
C18	Cochin Jewish	Jewish 3	051-234	12172-12308-12372-12705-15214	U2c	Present Study
C19	Cochin Jewish	Jewish 3	051-092-129C-362	152-204-217-508-12308-12372-12557-12705-15018	U2e	Present Study
C20	Cochin Jewish	Jewish 3	257-266-304-309-325-335-356	14990-12705-15385	R5a2b	Present Study
C21	Cochin Jewish	Jewish 3	051-092-129C-183C-189C-362	152-204-217-508-12308-12372-12557-12705-15018	U2e	Present Study
C22	Cochin Jewish	Jewish 3	182C-183C-189-249	73-146-195-263-459-12308-12372-12705-15954C	U1	Present Study
C23	Cochin Jewish	Jewish 3	129-172-234-274-309-362	73-195A-263-523d-525d-709-12285-12705-14990-15326-15385	R6a1a	Present Study
C24	Cochin Jewish	Jewish 3	051-206C-230-304-311-519	73-146-195-263-459-12308-12357-12372-12705-15049-15940	U2a1	Present Study
C25	Cochin Jewish	Jewish 3	040-295-311	480-10142-12207-12705	R30b	Present Study
C26	Cochin Jewish	Jewish 3	051-206C-230-304-311-519	12308-12357-12372-12705-15049-15940	U2a1	Present Study
C27	Cochin Jewish	Jewish 3	266-304-309-325-356	73-152-263-523d-525d-14990-12705-15385	R5a2b	Present Study
C28	Cochin Jewish	Jewish 3	067-182-183-327A	12705-15218	HV1a3	Present Study
C29	Cochin Jewish	Jewish 3	182C-183C-189-249	12308-12372-12705-15954C	U1	Present Study
C30	Cochin Jewish	Jewish 3	051-187-189-206C	12308-12372-12705-15049	U2a1	Present Study
C31	Cochin Jewish	Jewish 3	129-172-234-266-274-309-362	12285-12705-14990-15326-15385	R6a1a	Present Study
C32	Cochin Jewish	Jewish 3	129-234-266-309-362	12285-12705-15326-15385	R6a1a	Present Study
C33	Cochin Jewish	Jewish 3	051-172-234-274-309-362	12285-12705-14990-15326-15385	R6a1a	Present Study
C34	Cochin Jewish	Jewish 3	051-187-189-206C	12308-12372-12705-15049	U2a1	Present Study
C35	Cochin Jewish	Jewish 3	126-185-223-519	73-195-263-482-489-10398-10400-15018-15043-15301	M3	Present Study
C36	Cochin Jewish	Jewish 3	223	10398-10400-15043-15301	M4	Present Study
C37	Cochin Jewish	Jewish 3	951-126-185-223-519	73-195-263-482-489-10398-10400-15018-15043-15301	M3	Present Study
C38	Cochin Jewish	Jewish 3	257-266-304-309-325-335-356	12705-15385	R5a2b	Present Study
H1	Cochin Jewish	Jewish 3	172-183C-189-274-278-319	73-152-182-195-447G-523d-525d-10398-10400-15043-15301-15670	M2a1b	Present Study
H2	Cochin Jewish	Jewish 3	187-223-270-274-291-319-320-352-362	73-143-195-263-337-447G-10398-10400-15043-15301-15497-15670	M2a1a	Present Study
H3	Cochin Jewish	Jewish 3	129-172-223-234-266-274-309-362	12285-12705-15326-15385	R6a1a	Present Study
H4	Cochin Jewish	Jewish 3	114A-129-223-362	12285	R6a	Present Study
H5	Cochin Jewish	Jewish 3	114A-129-223-362	12285	R6a	Present Study
H6	Cochin Jewish	Jewish 3	051-126-185-223	73-195-263-482-489-10398-10400-15018-15043-15301	M3	Present Study
H7	Cochin Jewish	Jewish 3	129-172-234-266-274-309-362	12285-12705-15326-15385	R6a1a	Present Study
H8	Cochin Jewish	Jewish 3	126-154-223-224-311	73-146-195-263-482-10398-10400	M3	Present Study
H9	Cochin Jewish	Jewish 3	223-234-362	73-195-263-482-10398-10400-15043-15301	M35	Present Study
H10	Cochin Jewish	Jewish 3	129-172-234-266-274-309-362	73-146-195-263-459-12285-12705-14990-15326-15385	R6a1a	Present Study
H11	Cochin Jewish	Jewish 3	129-172-234-266-274-309-362	73-146-195-263-459-12285-12705-14990-15326-15385	R6a1a	Present Study
H12	Cochin Jewish	Jewish 3	129-172-234-266-274-278-309-362	73-146-195-263-459-12285-12705-15326-15385	R6a1a	Present Study
H13	Cochin Jewish	Jewish 3	257-266-304-309-325-335-356	12705-15385	R5a2b	Present Study
H14	Cochin Jewish	Jewish 3	266-304-309-325-356	73-152-189-246-523d-525d-12705-15385	R5a2b	Present Study
H15	Cochin Jewish	Jewish 3	126-185-223-519	73-195-263-482-489-10398-10400-15018-15043-15301	M3	Present Study
H16	Cochin Jewish	Jewish 3	093-129-362	12133-12285-15043-15130-15253-15301-15670	R6a2	Present Study
H17	Cochin Jewish	Jewish 3	129-172-234-266-274-309-362	73-228-263-12285-12705-14990-15326-15385	R6a1a	Present Study
H18	Cochin Jewish	Jewish 3	069-126-185-222-519	10084-12705-15951	J1c8	Present Study
H19	Cochin Jewish	Jewish 3	111-187-189-223-274-319-320-362	73-143-195-263-337-447G-489-10398-10400-15018-15043-15130-15253-15301-15670	M2b	Present Study
H20	Cochin Jewish	Jewish 3	092-111-187-189-223-274-319-320-362	73-143-195-263-337-447G-489-10398-10400-15018-15043-15130-15253-15301-15670	M2b	Present Study
H21	Cochin Jewish	Jewish 3	051-126-185-223-519	73-195-263-482-489-10398-10400-15018-15043-15301	M3	Present Study
H22	Cochin Jewish	Jewish 3	126-154-223-224	10398-10400-15043-15301	M3b	Present Study
H23	Cochin Jewish	Jewish 3	140-183C-189-266A	73-195-263-4829bp del-10398-12705-15235	B5a	Present Study
H24	Cochin Jewish	Jewish 3	111-144-223-224-311	9545	N5b	Present Study
H25	Cochin Jewish	Jewish 3	051-092-129C-183C-189-362	152-204-217-508-12308-12372-12557-12705-15018	U2e	Present Study
H26	Cochin Jewish	Jewish 3	069-126-185-222-519	10084-12705-15951	J1c8	Present Study

H27	Cochin Jewish	Jewish 3	140-183C-189-266A	9bp del-10398-12705-15235	B5a	Present Study
H29	Cochin Jewish	Jewish 3	129-172-234-266-274-309-362	73-195-263-482-12285-12705-15326-15385	R6a1a	Present Study
H30	Cochin Jewish	Jewish 3	051-093-153-182C-183C-189-218-263-292	73-146-195-263-459-12705	R31b	Present Study
H31	Cochin Jewish	Jewish 3	51	73-263-567-12070-12172-12308-12372-15148	U1	Present Study
H32	Cochin Jewish	Jewish 3	051-234	12308-12372-12705	U2c	Present Study
H33	Cochin Jewish	Jewish 3	051-093-129-234-311-362	73-146-152-263-709-12172-12308-12372-12705	U2c1b	Present Study
H34	Cochin Jewish	Jewish 3	182C-183C-189-249	12308-372-557-15954C	U1	Present Study
H35	Cochin Jewish	Jewish 3	051-092-129C-183C-189-362	152-204-217-508-12308-12372-12557-12705-15018	U2e	Present Study
H37	Cochin Jewish	Jewish 3	051-129C-183C-189-362	152-204-217-508-12308-12372-12557-12705-15018	U2e	Present Study
H38	Cochin Jewish	Jewish 3	093-129-362	12133-12285-12705	R6a2	Present Study
H39	Cochin Jewish	Jewish 3	129-172-234-266-274-309-362	12285-12705-14990-15326-15385	R6a1a	Present Study
H40	Cochin Jewish	Jewish 3	069-126-222	10084-12705	J1c8	Present Study
H42	Cochin Jewish	Jewish 3	270-274-291-319-352	73-195-204-447G-10398-10400-15043-15301-15497-15670	M2a1a	Present Study
H43	Cochin Jewish	Jewish 3	270-274-291-319-352	73-195-204-447G-10398-10400-15043-15301-15497-15670	M2a1a	Present Study
H44	Cochin Jewish	Jewish 3	223	73-199-263-10398-10400-15043-15301-15924	M35a	Present Study
H45	Cochin Jewish	Jewish 3	270-274-291-319-352	73-195-204-447G-10398-10400-15043-15301-15497-15670	M2a1a	Present Study
H46	Cochin Jewish	Jewish 3	172-278	73-234-263-523-525d-10398-10400-15043-15055-15241-15301-15562	M33a2	Present Study
H47	Cochin Jewish	Jewish 3	223-295	10398-10400-10556	M37e	Present Study
h36b	Cochin Jewish	Jewish 3	172-183C-189-274-319-320	10398-10400-15043-15301-15670	M2a1b	Present Study
32b	Cochin Jewish	Jewish 3	129-172-234-266-274-309-362	12285-12705-15326-15385	R6a1a	Present Study
B	Cochin Jewish	Jewish 3	069-274-362	10398-10400-12007-15043-15301-15431	M30c1a1	Present Study
J89	Cochin Jewish	Jewish 3	223	10398-10400-12007-15043-15301-15431	M30	Present Study
DB43	Cochin Jewish	Jewish 3	051-206C-215-230-255-304-311	12308-12372-12414-12705-15940	U2a1	Present Study
DB68	Cochin Jewish	Jewish 3	223-290-311-319-362	663	A	Present Study
DB46	Cochin Jewish	Jewish 3	150-185-223-260	249d1-0398-10400-12241del-15043-15261-15301-15326-15486	Z1	Present Study
DB51	Cochin Jewish	Jewish 3	093-223-290-293C-319-362	663	A	Present Study
	Cochin Jewish	Jewish 3	111-184-185-186-189-223-295	10398-10400-10556-12007-15043-15301	M37e	Present Study
S2	Cochin Jewish	Jewish 3	311	482-10398-10400-15043-15301	M3	Present Study
S106	Cochin Jewish	Jewish 3	309-318T	12172-12308-12372	U7	Present Study
S69	Cochin Jewish	Jewish 3	051-092-129C-183C-189-362	152-204-217-508-12308-12372-12557-12705-15018	U2e	Present Study
S67	Cochin Jewish	Jewish 3	051-092-129C-183C-189-362	152-204-217-508-12308-12372-12557-12705-15018	U2e	Present Study
S88	Cochin Jewish	Jewish 3	051-206C-230-304-311	12308-12357-12372-12705-15049	U2a1	Present Study
S22	Cochin Jewish	Jewish 3	126-185-223	73-195-263-482-489-10398-10400-15018-15043-15301	M3	Present Study
S54	Cochin Jewish	Jewish 3	182C-183C-189-249	12308-372-705-15217-15954C	U1	Present Study
S6	Cochin Jewish	Jewish 3	182C-183C-189-249	12308-12357-12372-12705-15954C	U1	Present Study
SZ	Cochin Jewish	Jewish 3	040-183C-189-311	480-10142-12207-12705	R30b	Present Study
Z4	Cochin Jewish	Jewish 3	067-093-183C-189-327A	12705-15218	HV1a3	Present Study
S82	Cochin Jewish	Jewish 3	051-092-129C-183C-189-362	152-204-217-508-12308-12372-12557-12705-15018	U2e	Present Study
S53	Cochin Jewish	Jewish 3	093-129-362	12285-12705	R6a	Present Study
S72	Cochin Jewish	Jewish 3	111-187-223-274-319-320-362	10398-10400-12172-15043-15253-15301-15670	M2a1a1a1	Present Study
S46	Cochin Jewish	Jewish 3	111-223	10398-10400-15043-15301	M4	Present Study
S103	Cochin Jewish	Jewish 3	126-223	482-10398-10400-15043-15301	M3	Present Study
S65	Cochin Jewish	Jewish 3	126-223	482-10398-10400-15043-15301	M3	Present Study
S85	Cochin Jewish	Jewish 3	126-223	482-10398-10400-15043-15301	M3	Present Study
S86	Cochin Jewish	Jewish 3	129-172-234-266-274-309-362	12285-12705	R6a1	Present Study
S76	Cochin Jewish	Jewish 3	129-172-234-266-274-309-362	12285-12705	R6a1	Present Study
S77	Cochin Jewish	Jewish 3	129-172	12285-12705	R6a1	Present Study
S45	Cochin Jewish	Jewish 3	172-278	12705-12714	R30a	Present Study
S73	Cochin Jewish	Jewish 3	172-234	12285-12705	R6a1	Present Study
S50	Cochin Jewish	Jewish 3	176-223-270-274-319-352	10398-10400-15018-15043-15301-15670	M2a1a1a1	Present Study
S4	Cochin Jewish	Jewish 3	181-309-318T	12172-12308-12372	U7	Present Study
SB28	Cochin Jewish	Jewish 3	182C-183C-189-249	12308-12372-12705-15217-15954C	U1	Present Study
83	Cochin Jewish	Jewish 3	184-223-362	10398-10400-12133-15043-15253-15301	M6	Present Study
S56	Cochin Jewish	Jewish 3	185-223-260-298	10398-10400-12241del-15043-15261-15301-15326-15486	Z1	Present Study
S84	Cochin Jewish	Jewish 3	223-519	10398-10400-15043-15301	M	Present Study
S51	Cochin Jewish	Jewish 3	223-519	10398-10400-15043-15301	M	Present Study
S52	Cochin Jewish	Jewish 3	223-519	10398-10400-15043-15301	M	Present Study
S87	Cochin Jewish	Jewish 3	257-266-304-309-325-335-356	12705-15385	R5a2b	Present Study
S55	Cochin Jewish	Jewish 3	257-266-304-309-325-335-356	12705-15385	R5a2b	Present Study
S8	Cochin Jewish	Jewish 3	318T	12172-12308-12372	U7	Present Study
kur1	Kurchian	Kurchian	169+C-173-223-189-223-274-311-319-320	263-316-447G-489-10398-10400	M2b	Present Study
kur2	Kurchian	Kurchian	169+C-173-223-189-223-274-311-319-320	263-316-447G-489-10398-10400	M2b	Present Study
kur3	Kurchian	Kurchian	169+C-173-223-189-223-274-311-319-320	263-316-447G-489-10398-10400	M2b	Present Study
kur4	Kurchian	Kurchian	169+C-173-223-189-223-274-311-319-320	263-316-447G-489-10398-10400	M2b	Present Study
kur5	Kurchian	Kurchian	169+C-173-223-189-223-274-311-319-320	263-316-447G-489-10398-10400	M2b	Present Study
kur6	Kurchian	Kurchian	169+C-173-223-189-223-274-311-319-320	263-316-447G-489-10322-10398-10400	M2b	Present Study
kur7	Kurchian	Kurchian	169+C-173-223-189-223-274-311-319-320	263-316-447G-489-10398-10400	M2b	Present Study
kur8	Kurchian	Kurchian	169+C-173-223-189-223-274-311-319-320	263-310+C-316-447G-489-10398-10400	M2b	Present Study
kur9	Kurchian	Kurchian	169+C-173-223-189-223-274-311-319-320	263-316-447G-489-10398-10400	M2b	Present Study
kur10	Kurchian	Kurchian	169+C-173-223-189-223-274-311-319-320	263-316-447G-489-10398-10400	M2b	Present Study
kur11	Kurchian	Kurchian	169+C-173-223-189-223-274-311-319-320	263-316-447G-489-10398-10400	M2b	Present Study
kur12	Kurchian	Kurchian	169+C-173-223-189-223-274-311-319-320	263-316-447G-489	M2b	Present Study
kur13	Kurchian	Kurchian	169+C-173-223-274-289-319-320-362	263-316-447G-489-10400-10463-10640	M2b	Present Study
kur14	Kurchian	Kurchian	169+C-173-223-274-289-319-320-362	263-316-447G-489-10398-10400	M2b	Present Study
kur15	Kurchian	Kurchian	169+C-173-223-189-223-274-311-319-320	263-316-447G-489-10322-10398-10400	M2b	Present Study
kur16	Kurchian	Kurchian	169+C-173-223-311-311-319	263-316-447G-489-10211t-10398-10400	M2b	Present Study
kur17	Kurchian	Kurchian	169+C-173-223-274-289-319-320-362	263-316-447G-489-523-10398-10400	M2b	Present Study
kur18	Kurchian	Kurchian	169+C-173-223-274-289-319-320-362	263-316-447G-489-10322-10398-10400	M2b	Present Study
kur19	Kurchian	Kurchian	169+C-173-223-274-289-319-320-362	263-316-447G-489-507-513C-10398-10400	M2b	Present Study
kur20	Kurchian	Kurchian	169+C-173-223-274-289-319-320-362	263-316-447G-489-523-10322-10398-10400	M2b	Present Study
kur21	Kurchian	Kurchian	169+C-173-223-274-289-319-320-362	263-316-447G-489-10398-10400	M2b	Present Study
kur22	Kurchian	Kurchian	169+C-173-223-274-289-319-320-362	263-316-447G-489-523-10398-10400	M2b	Present Study
kur23	Kurchian	Kurchian	169+C-173-223-274-289-319-320-362	258-263-316-447G-489-15301-15326-15670c10313-10398-10400-10463	M2b	Present Study
kur24	Kurchian	Kurchian	169+C-173-223-274-289-319-320-362	263-316-447G-48915301-15326-15670-10398-10400	M2b	Present Study
kur25	Kurchian	Kurchian	169+C-173-223-274-289-319-320-362	263-316-447G-489-523-10322-10398-10400-15301-15326-15670	M2b	Present Study
kur26	Kurchian	Kurchian	173-223-274-319-320	263-316-447G-489-523-10322-10398-1040015301-15326-15670	M2a1a	Present Study
kur27	Kurchian	Kurchian	173-223-274-319-320	195c-259-263-316-447G-489-10398-10400-15301-15326-15670-	M2a1a	Present Study
kur28	Kurchian	Kurchian	173-223-274-319-320	259-263-316-447G-10398-10400-489-15301-15326-15670	M2a1a	Present Study
kur29	Kurchian	Kurchian	173-223-274-319-320	259-263-316-447G-489-52315301-15326-15670-10398-10400	M2a1a	Present Study
kur30	Kurchian	Kurchian	173-223-274-319-320	259-263-316-447G-489-10398-10400-15301-15326-15670	M2a1a	Present Study
kur31	Kurchian	Kurchian	173-223-274-319-320	263-310-3196-447G-489-10398-10400-15301-15326-15670-10322	M2a1a	Present Study
kur32	Kurchian	Kurchian	223-274-319-320-390	259-263-316-447G-489-523-10398-10400-15326-15670	M2a1a	Present Study
kur33	Kurchian	Kurchian	173-223-274-319-320	259-263-316-447G-489-10398-10400-15326-15670	M2a1a	Present Study
kur34	Kurchian	Kurchian	173-223-274-319-320	259-263-316-447G-489-10398-10400-15326-15670	M2a1a	Present Study
kur35	Kurchian	Kurchian	173-223-274-319-320	263-316-447G-489-10398-10400-15326-15670	M2a1a	Present Study
kur36	Kurchian	Kurchian	173-223-274-319-320	259-263-316-447G-489-10398-10400-15670	M2a1a	Present Study
kur37	Kurchian	Kurchian	173-223-274-319-320	259-263-316-447G-489-10398-10400-15326-15670	M2a1a	Present Study
kur38	Kurchian	Kurchian	173-223-274-319-320	259-263-316-447G-489-10398-10400	M2a1a	Present Study
kur39	Kurchian	Kurchian	173-223-274-319-320	-259-263-316-447G-489-10398-10400	M2a1a	Present Study
kur40	Kurchian	Kurchian	173-223-274-319-320	259-263-316-447G-489-523-10398-10400	M2a1a	Present Study
kur41	Kurchian	Kurchian	173-223-274-319-320	259-263-316-447G-489-523-10398-10400	M2a1a	Present Study
kur42	Kurchian	Kurchian	173-223-274-319-320	259-263-316-447G-489-523-10322-10398-10400	M2a1a	Present Study
kur43	Kurchian	Kurchian	111-223-274-319-320	152c-182t-185(C)-10398-10400	M2a1a	Present Study
kur44	Kurchian	Kurchian	111-223	152-182-185-195-10398-10400-15301-15314-15326-15431	M30e	Present Study
kur45	Kurchian	Kurchian	111-223	152-182-185-195-10398-10400-15301-15314-15326-15431	M30e	Present Study
kur46	Kurchian	Kurchian	111-223	152-182-185-195-10398-10400-15301-15314-15326-15431	M30e	Present Study
kur47	Kurchian	Kurchian	111-223	152-182-185-195-10398-10400-15301-15314-15326-15431	M30e	Present Study
kur48	Kurchian	Kurchian	16111-16223	195A-259-263-316-489-523-10398-10400-15301-15314-15326-15431	M30	Present Study
kur49	Kurchian	Kurchian	16111-16223	195A-259-263-316-489-523-10398-10400-15301-15314-15326-15431	M30	Present Study

kur50	Kurchian	Kurchian	092c-111-223	152-182-185-195-10398-10400-15301-15314-15326-15431	M30e	Present Study
kur51	Kurchian	Kurchian	092-111-223	195A-263-316-489-523-10398-10400-15301-15314-15326-15431	M30	Present Study
kur52	Kurchian	Kurchian	092-111-223	152-182-185-195-10398-10400-15301-15314-15326-15431	M30d	Present Study
kur53	Kurchian	Kurchian	092-111-223	152-182-185-195-10398-10400-15301-15314-15326-15431	M30d	Present Study
kur54	Kurchian	Kurchian	111-223	195A-263-316-489-523-10322-10398-10400-15301-15314-15326-15431	M30	Present Study
kur55	Kurchian	Kurchian	092-111-223	195A-263-316-489-523-10322-10398-10400-15301-15314-15326-15431	M30	Present Study
kur56	Kurchian	Kurchian	111-223	195A-263-316-489-523-10322-10398-10400-15301-15314-15326-15431	M30	Present Study
kur57	Kurchian	Kurchian	111-223	195A-263-316-489-523-10398-10400-15326-15431	M30	Present Study
kur58	Kurchian	Kurchian	111-223	195A-263-316-489-523-10398-10400-15314-15326-15431	M30	Present Study
kur59	Kurchian	Kurchian	111-223	152-182-185-195-10398-10400-15314-15326-15431	M30d	Present Study
kur60	Kurchian	Kurchian	111-223	195A-259-263-10398-10400-15326-15431	M30	Present Study
kur61	Kurchian	Kurchian	111-223	152-182-185-195-10398-10400-15314-15326-15431	M30d	Present Study
kur62	Kurchian	Kurchian	111-223	195A-263-316-489-523-10398-10400-15314-15326-15431	M30	Present Study
kur63	Kurchian	Kurchian	111-223	195A-263-316-489-523-10398-10400-15314-15326-15431	M30	Present Study
kur64	Kurchian	Kurchian	092-111-223	146-152-185-195-10398-10400-15314-15326-15431	M30c	Present Study
kur65	Kurchian	Kurchian	111-223	195A-263-316-489-523-10398-10400-15314-15326-15431	M30	Present Study
kur66	Kurchian	Kurchian	111-223	195A-263-316-489-523-10398-10400-15326-15431	M30	Present Study
kur67	Kurchian	Kurchian	111-223	195A-263-316-489-523-10398-10400-15314-15326-15431	M30	Present Study
kur68	Kurchian	Kurchian	111-223	195A-263-316-489-523-10398-10400-15314-15326-15431	M30	Present Study
kur69	Kurchian	Kurchian	111-223	195A-263-316-489-523-10398-10400-15314-15326-15431	M30	Present Study
kur70	Kurchian	Kurchian	092-111-223	152-182-185-195-10322-10398-10400-15314-15326-15431	M30d	Present Study
kur71	Kurchian	Kurchian	092-111-223	195A-263-316-489-10398-10400-15314-15326-15431	M30	Present Study
kur72	Kurchian	Kurchian	092-111-223	152-182-185-195-10398-10400-15301-15314-15326-15431	M30d	Present Study
kur73	Kurchian	Kurchian	111-223	195A-263-316-489-523-10398-10400-15301-15314-15326-15431	M30	Present Study
kur74	Kurchian	Kurchian	111-223	152-182-185-195-10398-10400-15301-15314-15326-15431	M30d	Present Study
kur75	Kurchian	Kurchian	111-223	259-263-316+C-489-574+C-10398-10400-15301-15314-15326-15431	M30	Present Study
kur76	Kurchian	Kurchian	111-223	259-263-316-489-523-10398-10400-15301-15314-15326-15431	M30	Present Study
kur77	Kurchian	Kurchian	111-223	259-263-316-489-523-10398-10400-15301-15314-15326-15431	M30	Present Study
kur78	Kurchian	Kurchian	092-111-223	259-263-316-489-523-10398-10400-15301-15314-15326-15431	M30	Present Study
kur79	Kurchian	Kurchian	111-223	259-263-316-489-523-10398-10400-15301-15314-15326-15431	M30	Present Study
kur80	Kurchian	Kurchian	092-111-223	259-263-316-489-523-10398-10400-15301-15314-15326-15431	M30	Present Study
kur81	Kurchian	Kurchian	092-111-223	259-263-316-489-523-10398-10400-15301-15314-15326-15431	M30	Present Study
kur82	Kurchian	Kurchian	092-111-223	259-263-316-489-523-10398-10400-15301-15314-15326-15431	M30	Present Study
kur83	Kurchian	Kurchian	092-111-223	263-316-489-523-10398-10400-15301-15314-15326-15431	M30	Present Study
kur84	Kurchian	Kurchian	092-111-223	259-263-316-489-523-10398-10400-15301-15314-15326-15431	M30	Present Study
kur85	Kurchian	Kurchian	111-223	259-263-316-489-523-10398-10400-15326-15431	M30	Present Study
kur86	Kurchian	Kurchian	092-223-172c	215g-263-310-316-10398-10400-15314-15326-15431	M30	Present Study
kur87	Kurchian	Kurchian	111-223	259-263-316-489-523-10322-10398-10400-15314-15326-15431	M30	Present Study
kur88	Kurchian	Kurchian	111-223	259-263-316-489-523-10398-10400-15326-15431	M30	Present Study
kur89	Kurchian	Kurchian	092-111-223	259-263-316-489-523-10398-10400-15314-15326-15431	M30	Present Study
kur90	Kurchian	Kurchian	092-111-223	259-263-316-489-523-10398-10400-15314-15326-15431	M30	Present Study
kur91	Kurchian	Kurchian	092-111-223	263-316-489-523-10398-10400-15314-15326-15431	M30	Present Study
kur92	Kurchian	Kurchian	092-111-223	259-263-316-489-523-10398-10400-15314-15326-15431	M30	Present Study
kur93	Kurchian	Kurchian	129-134-213-223-240-298-362	259-263-316-447G-489	M30	Present Study
kur94	Kurchian	Kurchian	223-287-304	195A-263-316-489-523d	M30	Present Study
kur95	Kurchian	Kurchian	129-134-213-223-240-298-362	195-259-263-316-489-523	M30	Present Study
kur96	Kurchian	Kurchian	092-223-173-223-274-289-319-320	263-310-3196-447G-489-10322-10398-10400-15301-15326-15670	M2b	Present Study
4328	Ulladan	Ulladan	223-270-274-319-352	10398-10400-15043-15301-15670	M2a1	Present Study
4335	Ulladan	Ulladan	129-223	10398-10400-12477-15043-15301	M5	Present Study
4337	Ulladan	Ulladan	172-183C-189-278	12705-12714	R30a	Present Study
4338	Ulladan	Ulladan	129-223	10398-10400-12477-15043-15301	M5	Present Study
4341	Ulladan	Ulladan	093-223-270-274-319-352	10398-10400-15043-15301-15670	M2a1a1	Present Study
4342	Ulladan	Ulladan	093-223-270-274-319-352	10398-10400-15043-15301-15670	M2a1a1	Present Study
4344	Ulladan	Ulladan	129-223	10398-10400-12477-15043-15301	M5	Present Study
4346	Ulladan	Ulladan	093-223-270-274-319-352	10398-10400-15043-15301-15670	M2a1a1	Present Study
4348	Ulladan	Ulladan	093-223-270-274-319-352	10398-10400-15043-15301-15670	M2a1a1	Present Study
4350	Ulladan	Ulladan	172-183C-189-278	12705-12714	R30a	Present Study
4351	Ulladan	Ulladan	172-183C-189-278	12705-12714	R30a	Present Study
4353	Ulladan	Ulladan	093-223-270-274-319-352	10398-10400-15043-15301-15670	M2a1a1	Present Study
4354	Ulladan	Ulladan	172-183C-189-278	12705-12714	R30a	Present Study
4355	Ulladan	Ulladan	129-223	10398-10400-12477-15043-15301	M5	Present Study
4357	Ulladan	Ulladan	172-183C-189-278	12705-12714	R30a	Present Study
4358	Ulladan	Ulladan	093-223-270-274-319-352	10398-10400-15043-15301-15670	M2a1a1	Present Study
4359	Ulladan	Ulladan	129-223	10398-10400-12477-15043-15301	M5	Present Study
4360	Ulladan	Ulladan	129-223	10398-10400-12477-15043-15301	M5	Present Study
4362	Ulladan	Ulladan	129-223	10398-10400-12477-15043-15301	M5	Present Study
4363	Ulladan	Ulladan	172-183C-189-278	12705-12714	R30a	Present Study
4364	Ulladan	Ulladan	172-183C-189-278	12705-12714	R30a	Present Study
4365	Ulladan	Ulladan	172-183C-189-278	12705-12714	R30a	Present Study
4366	Ulladan	Ulladan	129-223	10398-10400-12477-15043-15301	M5	Present Study
4367	Ulladan	Ulladan	129-223	10398-10400-12477-15043-15301	M5	Present Study
4376	Ulladan	Ulladan	129-223	10398-10400-12477-15043-15301	M5	Present Study
D4	Ulladan	Ulladan	223-262-311	10398-10400-15043-15301	M36c	Present Study
D6	Ulladan	Ulladan	223	10398-10400-15043-15301-15431	M30	Present Study
J38E	Ulladan	Ulladan	126-223	482-10398-10400-15043-15301	M3	Present Study
4227	Malayan	Malayan	223-519	10398-10400-15043-15301	M	Present Study
4251	Malayan	Malayan	124-179-183C-189-223-294	10398-10400-15043-15301	M3c1b1	Present Study
4255	Malayan	Malayan	126-183C-189	482-10398-10400-15043-15301	M3	Present Study
4260	Malayan	Malayan	223	10398-10400-15043-15301	M4	Present Study
4267	Malayan	Malayan	093-174-223	10398-10400-15043-15301	M	Present Study
4271	Malayan	Malayan	223	10398-10400-15043-15301	M4	Present Study
4277	Malayan	Malayan	223	10398-10400-15043-15301-15431	M30	Present Study
4279	Malayan	Malayan	223	10398-10400-15043-15301-15431	M30	Present Study
4280	Malayan	Malayan	126-223	482-10398-10400-15043-15301	M3	Present Study
4284	Malayan	Malayan	223-262-311	10398-10400-15043-15301	M36c	Present Study
4286	Malayan	Malayan	223-262-311	10398-10400-15043-15301	M36c	Present Study
4290	Malayan	Malayan	223	10398-10400-15043-15301	M4	Present Study
4292	Malayan	Malayan	113C-129-223-262	10398-10400	M36c	Present Study
4293	Malayan	Malayan	113C-223-262-311	10398-10400	M36c	Present Study
4294	Malayan	Malayan	124-179-183C-189-223-294	10398-10400-15043-15301	M3c1b1	Present Study
4297	Malayan	Malayan	172-278	10398-10400-15043-15055-15241-15301-15562	M33a2	Present Study
4301	Malayan	Malayan	051-069-168-249-320-324-330-342	12308-12372-12705-15217	U1	Present Study
4304	Malayan	Malayan	111-124-148-177	12714	R30	Present Study
4310	Malayan	Malayan	184-223-311	10398-10400	M4	Present Study
4311	Malayan	Malayan	223	10398-10400-15043-15301	M4	Present Study
4314	Malayan	Malayan	223	10398-10400-15043-15301	M4	Present Study
4315	Malayan	Malayan	223	10398-10400-15043-15301-15431	M30	Present Study
4318	Malayan	Malayan	223	10398-10400-15043-15301	M4	Present Study
4320	Malayan	Malayan	126-223-347	10398-10400-15043-15301	M	Present Study
4322	Malayan	Malayan	126-223	482-10398-10400-15043-15301	M3	Present Study
4325	Malayan	Malayan	182C-183C-189	15385	R5a1	Present Study
4326	Malayan	Malayan	076-182C-183C-189-230-356	15285	U4	Present Study
P01	Paniya	Paniya	223-274-319	10398-10400-15043-15301-15670	M2a1	Present Study
P02	Paniya	Paniya	126-223	482-10398-10400-15043-15301	M3	Present Study
P03	Paniya	Paniya	158T-223-274-319	10398-10400-15043-15301-15670	M2a1	Present Study
P04	Paniya	Paniya	126-223	482-10398-10400-15043-15301	M3	Present Study
P10	Paniya	Paniya	126-145-223	482-10398-10400-15043-15301	M3	Present Study
P11	Paniya	Paniya	111A-129-223	10398-10400-15043-15301	M5	Present Study
P12	Paniya	Paniya	126-223	482-10398-10400-15043-15301	M3	Present Study

P13	Paniya	Paniya	126-223	482-10398-10400-15043-15301	M3	Present Study
P14	Paniya	Paniya	069-129-274-318T	12172-12308-12372	U7	Present Study
P15	Paniya	Paniya	189	12705	R	Present Study
P16	Paniya	Paniya	223-234-262-286	10398-10400-15043-15301	M36c	Present Study
P17	Paniya	Paniya	172-278	10398-10400-15043-15055-15241-15301-15562	M33a2	Present Study
P18	Paniya	Paniya	126-223	482-10398-10400-15043-15301	M3	Present Study
P19	Paniya	Paniya	126-223	482-10398-10400-15043-15301	M3	Present Study
P20	Paniya	Paniya	189-371	12705	R	Present Study
P22	Paniya	Paniya	223-274-319	10398-10400-15043-15301-15670	M2a1	Present Study
P23	Paniya	Paniya	126-223	482-10398-10400-15043-15301	M3	Present Study
P24	Paniya	Paniya	126-223	482-10398-10400-15043-15301	M3	Present Study
P25	Paniya	Paniya	223-274-319	10398-10400-15043-15301-15670	M2a1	Present Study
P26	Paniya	Paniya	223-234-262	10398-10400-15043-15301	M36c	Present Study
P27	Paniya	Paniya	172-278	10398-10400-15043-15055-15241-15301-15562	M33a2	Present Study
P29	Paniya	Paniya	126-223	482-10398-10400-15043-15301	M3	Present Study
P30	Paniya	Paniya	093-223	10398-10400-15043-15301-15924	M35a	Present Study
P31	Paniya	Paniya	129-362	12285-12705	R6a	Present Study
P32	Paniya	Paniya	126-223	482-10398-10400-15043-15301	M3	Present Study
P33	Paniya	Paniya	126-223	482-10398-10400-15043-15301	M3	Present Study
P34	Paniya	Paniya	093-223	10398-10400-15043-15301-15924	M35a	Present Study
P35	Paniya	Paniya	172-278	10398-10400-15043-15055-15241-15301-15562	M33a2	Present Study
P36	Paniya	Paniya	189-239A	12705	R	Present Study
P39	Paniya	Paniya	129-148-362	12285-12705	R6a	Present Study
P40	Paniya	Paniya	093-223	10398-10400-15043-15301-15924	M35a	Present Study
AY 2	Adiyan	Adiyan	093- 129- 223	15043-15301-15355-15924	M5	Present Study
AY 3	Adiyan	Adiyan	093- 129- 223	15043-15301-15355-15924	M5	Present Study
AY 9	Adiyan	Adiyan	093- 129- 223	15043-15301-15355-15924	M5	Present Study
AY 11	Adiyan	Adiyan	093- 129- 223	15043-15301-15355-15924	M5	Present Study
AY 15	Adiyan	Adiyan	093- 129- 223	3714-15043-15301-15355-15924	M5	Present Study
AY 17	Adiyan	Adiyan	093- 129- 223	15043-15301-15670	M5	Present Study
AY 18	Adiyan	Adiyan	093- 129- 223	15043-15301-15355-15924	M5a	Present Study
AY 19	Adiyan	Adiyan	093- 129- 223	15043-15301-15355-15924	M5a	Present Study
AY 20	Adiyan	Adiyan	093- 129- 223	15924	M5	Present Study
AY 29	Adiyan	Adiyan	093- 129- 223	15043-15301-15355-15924	M5	Present Study
AY 33	Adiyan	Adiyan	093- 129- 223	15043-15301-15355-15924	M5	Present Study
AY 58	Adiyan	Adiyan	093- 129- 223	15043-15301-15355-15924	M5	Present Study
AY 59	Adiyan	Adiyan	093- 129- 223	15043-15301-15355-15924	M5a	Present Study
AY 60	Adiyan	Adiyan	093- 223- 129	15043-15301-15355-15924	M5a	Present Study
K01	Kuruman	Kuruman	093-223-319	10398-10400-15043-15301-15670	M2a1	Present Study
K02	Kuruman	Kuruman	223-319	10398-10400-15043-15301-15670	M2a1	Present Study
K04	Kuruman	Kuruman	093-223	10398-10400-15043-15301-15924	M35a	Present Study
K05	Kuruman	Kuruman	093-223-319	10398-10400-15043-15301-15670	M2a1	Present Study
K06	Kuruman	Kuruman	093-223	10398-10400-15043-15301-15924	M35a	Present Study
K07	Kuruman	Kuruman	093-223-319	10398-10400-15043-15301-15670	M2a1	Present Study
K08	Kuruman	Kuruman	223-319	10398-10400-15043-15301-15670	M2a1	Present Study
K09	Kuruman	Kuruman	093-223-319	10398-10400-15043-15301-15670	M2a1	Present Study
K10	Kuruman	Kuruman	093-223-319	10398-10400-15043-15301-15670	M2a1	Present Study
K11	Kuruman	Kuruman	093-223-319	10398-10400-15043-15301-15670	M2a1	Present Study
K13	Kuruman	Kuruman	093-223-319	10398-10400-15043-15301-15670	M2a1	Present Study
K15	Kuruman	Kuruman	093-223-319	10398-10400-15043-15301-15670	M2a1	Present Study
K16	Kuruman	Kuruman	093-223-319	10398-10400-15043-15301-15670	M2a1	Present Study
K17	Kuruman	Kuruman	223-319	10398-10400-15043-15301-15670	M2a1	Present Study
K18	Kuruman	Kuruman	093-223-319	10398-10400-15043-15301-15670	M2a1	Present Study
K19	Kuruman	Kuruman	093-223-319	10398-10400-15043-15301-15670	M2a1	Present Study
K21	Kuruman	Kuruman	051-092-111-129-223	10398-10400	M5	Present Study
K22	Kuruman	Kuruman	093-223-319	10398-10400-15043-15301-15670	M2a1	Present Study
K23	Kuruman	Kuruman	093-223-319	10398-10400-15043-15301-15670	M2a1	Present Study
K24	Kuruman	Kuruman	093-223-319	10398-10400-15043-15301-15670	M2a1	Present Study
K25	Kuruman	Kuruman	223-274-319	10398-10400-15043-15301-15670	M2a1	Present Study
K26	Kuruman	Kuruman	093-223-319	10398-10400-15043-15301-15670	M2a1	Present Study
K27	Kuruman	Kuruman	223-319	10398-10400-15043-15301-15670	M2a1	Present Study
K28	Kuruman	Kuruman	093-223-319	10398-10400-15043-15301-15670	M2a1	Present Study
K29	Kuruman	Kuruman	093-223-319	10398-10400-15043-15301-15670	M2a1	Present Study
K30	Kuruman	Kuruman	129-362	12285-12705	R6a	Present Study
K31	Kuruman	Kuruman	223	10398-10400-15043-15301-15431	M30	Present Study
NKN02	Kattunaiken	Kattunaiken	223-239-298-327	10398-10400-15043-15301	C4a4a	Present Study
NKN03	Kattunaiken	Kattunaiken	223	10398-10400-15043-15301-15431	M30	Present Study
NKN03	Kattunaiken	Kattunaiken	189-223-274-295-319-320	10398-10400-15043-15301-15670	M2a1	Present Study
NKN06	Kattunaiken	Kattunaiken	223-239-298-327-357	10398-10400-15043-15301	C4a4a	Present Study
NKN07	Kattunaiken	Kattunaiken	223-519	10398-10400-15043-15301	M	Present Study
NKN10	Kattunaiken	Kattunaiken	223-239-298-327-357	10398-10400-15043-15301	C4a4a	Present Study
NKN10	Kattunaiken	Kattunaiken	223-239-298-327-357	10398-10400-15043-15301	C4a4a	Present Study
NKN11	Kattunaiken	Kattunaiken	223	10398-10400-15043-15301	M4	Present Study
NKN11	Kattunaiken	Kattunaiken	223	10398-10400-15043-15301-15431	M30	Present Study
NKN13	Kattunaiken	Kattunaiken	223-239-298-327	10398-10400-15043-15301	C4a4a	Present Study
NKN15	Kattunaiken	Kattunaiken	136-183C-223-274-319-320	447G-489-10398-10400	M2b	Present Study
NKN15	Kattunaiken	Kattunaiken	189-223-274-319-320	10398-10400-15043-15301-15670	M2a1	Present Study
NKN17	Kattunaiken	Kattunaiken	129-362	12285-12705	R6a	Present Study
NKN21	Kattunaiken	Kattunaiken	172-278	10398-10400-15043-15055-15241-15301-15562	M33a2	Present Study
NKN22	Kattunaiken	Kattunaiken	136-183C-223-274-319-320	447G-489-10398-10400	M2b	Present Study
NKN23	Kattunaiken	Kattunaiken	182C-183C-189	12308-12372-12705-15217-15954C	U1a3	Present Study
NKN24	Kattunaiken	Kattunaiken	223	10398-10400-15043-15301-15431-15924	M30	Present Study
NKN28	Kattunaiken	Kattunaiken	183C-189-223-274-319-320	10398-10400	M2a1a1	Present Study
NKN28	Kattunaiken	Kattunaiken	183C-189-223-274-319-320	10398-10400	M2a1a1	Present Study
NKN30	Kattunaiken	Kattunaiken	223	10398-10400-15043-15301-15431	M30	Present Study
NKN31	Kattunaiken	Kattunaiken	172-278	10398-10400-15043-15055-15241-15301-15562	M33a2	Present Study
NKN31	Kattunaiken	Kattunaiken	172-278	10398-10400-15043-15055-15241-15301-15562	M33a2	Present Study
NKN32	Kattunaiken	Kattunaiken	172-278	10398-10400-15043-15055-15241-15301-15562	M33a2	Present Study
NKN33	Kattunaiken	Kattunaiken	172-278	10398-10400-15043-15055-15241-15301-15562	M33a2	Present Study
NKN34	Kattunaiken	Kattunaiken	223	10398-10400-15043-15301	M4	Present Study
NKN35	Kattunaiken	Kattunaiken	183C-223-274-319-320	10398-10400-15043-15301-15670	M2a1	Present Study
NKN35	Kattunaiken	Kattunaiken	189-223-274-319-320-519	10398-10400-15043-15301-15670	M2a1	Present Study
NKN37	Kattunaiken	Kattunaiken	136-183C-223-274-319-320	447G-489-10398-10400	M2b	Present Study
NKN37	Kattunaiken	Kattunaiken	136-183C-223-274-319-320	447G-489-10398-10400	M2b	Present Study
NKN37	Kattunaiken	Kattunaiken	183C-189-223-274-319-320-519	10398-10400	M2a1a1	Present Study
NKN37	Kattunaiken	Kattunaiken	183C-189-223-274-319-320-519	10398-10400	M2a1a1	Present Study
NKN38	Kattunaiken	Kattunaiken	223	10398-10400-15043-15301-15431	M30	Present Study
NKN39	Kattunaiken	Kattunaiken	223	10398-10400-15043-15301	M4	Present Study
NKN41	Kattunaiken	Kattunaiken	223-239-298-327-357	10398-10400-15043-15301	C4a4a	Present Study
NKN42	Kattunaiken	Kattunaiken	223	10398-10400-15043-15301-15431	M30	Present Study
NKN43	Kattunaiken	Kattunaiken	124-179-183C-189-223-294	10398-10400-15043-15301	M3c1b1	Present Study
NKN44	Kattunaiken	Kattunaiken	223-274-319-320	15018-15043-15301-15670	M2b	Present Study
NKN44	Kattunaiken	Kattunaiken	124-179-183C-189-223-294	10398-10400-15043-15301	M3c1b1	Present Study
NKN45	Kattunaiken	Kattunaiken	124-179-183C-249	12308-12357-12372-12705	U1	Present Study
NKN45	Kattunaiken	Kattunaiken	124-179-183C-189-223-294	10398-10400-15043-15301	M3c1b1	Present Study
NKN45	Kattunaiken	Kattunaiken	124-179-183C-249-519	12308-12357-12372-12705	U1	Present Study
NKN45	Kattunaiken	Kattunaiken	183C-189-249-519	12308-12372	U1	Present Study
NKN46	Kattunaiken	Kattunaiken	223	10398-10400-15043-15301-15431	M30	Present Study
NKN47	Kattunaiken	Kattunaiken	136-183C-223-274-319-320	447G-489-10398-10400	M2b	Present Study

NKN47	Kattunaiken	Kattunaiken	189-223-519	10398-10400-15043-15301	M	Present Study
NKN48	Kattunaiken	Kattunaiken	189	12705	R	Present Study
NKN48	Kattunaiken	Kattunaiken	189-223-294-519	10398-10400-15043-15301	M3c1b1	Present Study
NKN50	Kattunaiken	Kattunaiken	124-179-183C-189-223-294	10398-10400-15043-15301	M3c1b1	Present Study
NKN51	Kattunaiken	Kattunaiken	223-274-319-320	15043-15301-15670	M2b	Present Study
NKN51	Kattunaiken	Kattunaiken	124-179-183C-189-223-294	10398-10400-15043-15301	M3c1b1	Present Study
NKN51	Kattunaiken	Kattunaiken	179-183C-189-223-274-319-320-519	10398-10400	M2a1a1	Present Study
NKN51	Kattunaiken	Kattunaiken	179-183C-189-223-274-319-320-519	10398-10400	M2a1a1	Present Study
NKN52	Kattunaiken	Kattunaiken	223	10398-10400-15043-15301	M4	Present Study
NKN52	Kattunaiken	Kattunaiken	223-519	10398-10400-15043-15301	M	Present Study
NKN52	Kattunaiken	Kattunaiken	223-519	10398-10400-15043-15301	M	Present Study
NKN53	Kattunaiken	Kattunaiken	223	10398-10400-15043-15301	M4	Present Study
NKN54	Kattunaiken	Kattunaiken	223-239-298-327-357	10398-10400-15043-15301	C4a4a	Present Study
NKN56	Kattunaiken	Kattunaiken	223	10398-10400-15043-15301-15431	M30	Present Study
NKN57	Kattunaiken	Kattunaiken	189-223-294	10398-10400-15043-15301	M3c1b1	Present Study
NKN58	Kattunaiken	Kattunaiken	223	10398-10400-15043-15301-15431	M30	Present Study
NKN61	Kattunaiken	Kattunaiken	223	10398-10400-15043-15301-15431	M30	Present Study
NKN62	Kattunaiken	Kattunaiken	189	12705	R	Present Study
NKN62	Kattunaiken	Kattunaiken	189-230-356-519	15285	U4	Present Study
NKN62	Kattunaiken	Kattunaiken	183C-189-230-356	15385	R5a1	Present Study
NKN63	Kattunaiken	Kattunaiken	223	10398-10400-15043-15301-15431	M30	Present Study
NKN64	Kattunaiken	Kattunaiken	223	10398-10400-15043-15301	M4	Present Study
NKN70	Kattunaiken	Kattunaiken	223	10398-10400-15043-15301-15431	M30	Present Study
NKN71	Kattunaiken	Kattunaiken	093-223-234-262	10398-10400-15043-15301-15431	M30	Present Study
NKN72	Kattunaiken	Kattunaiken	223	10398-10400-15043-15301-15431	M30	Present Study
NKN73	Kattunaiken	Kattunaiken	126-223	482-10398-10400-15043-15301	M3	Present Study
NKN74	Kattunaiken	Kattunaiken	126-223	482-10398-10400-15043-15301	M3	Present Study
NKN75	Kattunaiken	Kattunaiken	223	10398-10400-15043-15301	M4	Present Study
NKN76	Kattunaiken	Kattunaiken	223	10398-10400-15043-15301-15431	M30	Present Study
NKN77	Kattunaiken	Kattunaiken	093-223-234-262	10398-10400-15043-15301-15431	M30	Present Study
NKN78	Kattunaiken	Kattunaiken	183C-189-223-274-319-320	477G-10398-10400	M2a1a1	Present Study
NKN79	Kattunaiken	Kattunaiken	223	10398-10400-15043-15301-15431	M30	Present Study
NKN79	Kattunaiken	Kattunaiken	189-223-274-319-320-519	10398-10400-15043-15301-15670	M2a1	Present Study
NKN80	Kattunaiken	Kattunaiken	124-179-183C-223-294	10398-10400-15043-15301	M3c1b1	Present Study
NKN83	Kattunaiken	Kattunaiken	124-179-183C-189-223-294	15285-15954	M40a	Present Study

Behar, D. M. *et al.* Counting the founders: the matrilineal genetic ancestry of the Jewishish Diaspora. *PLoS ONE.* **3**, e2062 (2008).

Supp. Table 6. The mtDNA haplogroups of Indian Jewish and their neighbouring local Indian populations

Population	n	A	B4c2	B5a	C4a4a	D5	F1	H13a2a1	H14	HV1a1	HV1a3	J1c2a	J1c8	K	K1a1b1a	K1a4d	M	M18
Jewish 1	34	-	-	-	-	-	-	0.059	0.029	-	-	-	-	-	-	-	0.147	-
Jewish 3	305	0.007	0.003	0.013	0.003	-	0.003	-	-	0.003	0.020	0.003	0.010	0.003	0.013	0.010	0.020	0.003
Jewish 4	45	-	-	-	-	0.022	-	-	-	-	-	-	-	-	-	-	0.044	-
Kurchian	96	-	-	-	-	-	-	-	-	-	-	-	-	-	-	-	-	-
Ulladan	28	-	-	-	-	-	-	-	-	-	-	-	-	-	-	-	-	-
Malayan	27	-	-	-	-	-	-	-	-	-	-	-	-	-	-	-	0.111	-
Paniya	31	-	-	-	-	-	-	-	-	-	-	-	-	-	-	-	-	-
Adiyan	14	-	-	-	-	-	-	-	-	-	-	-	-	-	-	-	-	-
Kuruman	27	-	-	-	-	-	-	-	-	-	-	-	-	-	-	-	-	-
Kattunaiken	79	-	-	-	0.089	-	-	-	-	-	-	-	-	-	-	-	0.051	-

Population	M2a1	M2a1a	M2a1a1	M2a1a1a1	M2a1b	M2b	M3	M30	M30	M30c	M30c1a1	M30d	M30e	M31a2	M33a2	M35	M35a	M35b	M36c
Jewish 1	-	-	-	-	-	-	-	-	-	-	0.118	-	-	-	-	-	-	-	-
Jewish 3	0.003	0.026	-	0.030	0.007	0.020	0.098	0.030	-	-	0.003	-	0.010	0.003	0.003	0.003	0.023	0.003	-
Jewish 4	-	-	-	-	-	-	-	0.022	-	-	-	-	-	-	-	-	-	-	-
Kurchian	-	0.188	-	-	-	0.271	-	0.406	-	0.010	-	0.073	0.052	-	-	-	-	-	-
Ulladan	0.036	-	0.214	-	-	-	0.036	-	0.036	-	-	-	-	-	-	-	-	-	0.036
Malayan	-	-	-	-	-	-	0.111	-	0.111	-	-	-	-	-	0.037	-	-	-	0.148
Paniya	0.129	-	-	-	-	-	0.387	-	-	-	-	-	-	-	0.097	-	0.097	-	0.065
Adiyan	-	-	-	-	-	-	-	-	-	-	-	-	-	-	-	-	-	-	-
Kuruman	0.815	-	-	-	-	-	-	-	0.037	-	-	-	-	-	-	-	0.074	-	-
Kattunaiken	0.063	-	0.089	-	-	0.089	0.025	-	0.215	-	-	-	-	-	0.063	-	-	-	-

Population	M37	M37e	M39	M39a1	M39b	M3b	M3c1b1	M4	M40a	M4a	M5	M5a	M5a1	M5a1b	M5a2	M6	M64	M6a1	N1a	N1a2	N5b
Jewish 1	-	-	0.088	0.441	-	-	-	-	-	-	-	-	-	-	-	-	0.029	-	-	-	-
Jewish 3	0.003	0.010	-	-	0.007	0.007	-	0.013	-	0.003	0.010	0.043	0.007	0.003	0.003	0.003	-	0.003	-	0.003	0.007
Jewish 4	-	-	-	-	-	-	-	-	-	-	-	-	0.267	-	-	-	0.178	-	0.044	-	-
Kurchian	-	-	-	-	-	-	-	-	-	-	-	-	-	-	-	-	-	-	-	-	-
Ulladan	-	-	-	-	-	-	-	-	-	-	0.357	-	-	-	-	-	-	-	-	-	-
Malayan	-	-	-	-	-	-	0.074	0.259	-	-	-	-	-	-	-	-	-	-	-	-	-
Paniya	-	-	-	-	-	-	-	-	-	-	0.032	-	-	-	-	-	-	-	-	-	-
Adiyan	-	-	-	-	-	-	-	-	-	-	0.714	0.286	-	-	-	-	-	-	-	-	-
Kuruman	-	-	-	-	-	-	-	-	-	-	0.037	-	-	-	-	-	-	-	-	-	-
Kattunaiken	-	-	-	-	-	-	0.101	0.089	0.013	-	-	-	-	-	-	-	-	-	-	-	-

Population	R	R30	R30a	R30b	R31b	R5	R5a1	R5a1a	R5a2b	R6a	R6a1	R6a1a	R6a2	R8	Z	Z1
Jewish 1	-	-	-	0.088	-	-	-	-	-	-	-	-	-	-	-	-
Jewish 3	0.007	-	0.013	0.016	0.007	-	-	-	0.059	0.020	0.016	0.075	0.026	0.010	0.007	0.020
Jewish 4	0.067	-	0.067	-	-	0.022	-	0.111	-	-	-	-	-	-	-	-
Kurchian	-	-	-	-	-	-	-	-	-	-	-	-	-	-	-	-
Ulladan	-	-	0.286	-	-	-	-	-	-	-	-	-	-	-	-	-
Malayan	-	0.037	-	-	-	-	0.037	-	-	-	-	-	-	-	-	-
Paniya	0.097	-	-	-	-	-	-	-	-	0.065	-	-	-	-	-	-
Adiyan	-	-	-	-	-	-	-	-	-	-	-	-	-	-	-	-
Kuruman	-	-	-	-	-	-	-	-	-	0.037	-	-	-	-	-	-
Kattunaiken	0.025	-	-	-	-	-	0.013	-	-	0.013	-	-	-	-	-	-

Population	U1	U1a	U1a3	U2a1	U2b2	U2c	U2c1b	U2e	U4	U5b1b	U7	W	Reference
Jewish 1	-	-	-	-	-	-	-	-	-	-	-	-	Behar et al. 2008
Jewish 3	0.049	-	-	0.033	0.013	0.013	0.003	0.056	0.003	0.010	0.043	-	Present Study
Jewish 4	0.111	0.022	-	-	-	-	-	-	-	-	-	0.022	Behar et al. 2008
Kurchian	-	-	-	-	-	-	-	-	-	-	-	-	Present Study
Ulladan	-	-	-	-	-	-	-	-	-	-	-	-	Present Study
Malayan	0.037	-	-	-	-	-	-	-	0.037	-	-	-	Present Study
Paniya	-	-	-	-	-	-	-	-	-	-	0.032	-	Present Study
Adiyan	-	-	-	-	-	-	-	-	-	-	-	-	Present Study
Kuruman	-	-	-	-	-	-	-	-	-	-	-	-	Present Study
Kattunaiken	0.038	-	0.013	-	-	-	-	-	0.013	-	-	-	Present Study

Behar, D. M. *et al.* Counting the founders: the matrilineal genetic ancestry of the Jewishish Diaspora. *PLoS ONE.* **3**, e2062 (2008).
